# Integrated omics profiling reveals novel patterns of epigenetic programming in cancer-associated myofibroblasts

**DOI:** 10.1093/carcin/bgz001

**Published:** 2019-01-08

**Authors:** Hanna Najgebauer, Triantafillos Liloglou, Puthen V Jithesh, Olivier T Giger, Andrea Varro, Christopher M Sanderson

**Affiliations:** 1 Department of Cellular and Molecular Physiology, University of Liverpool, Liverpool, UK; 2 Department of Molecular and Clinical Cancer Medicine, University of Liverpool, Liverpool, UK; 3 Department of Medicine, University of Szeged, Szeged, Hungary

## Abstract

There is increasing evidence that stromal myofibroblasts play a key role in the tumour development however, the mechanisms by which they become reprogrammed to assist in cancer progression remain unclear. As cultured cancer-associated myofibroblasts (CAMs) retain an ability to enhance the proliferation and migration of cancer cells *in vitro*, it is possible that epigenetic reprogramming of CAMs within the tumour microenvironment may confer long-term pro-tumourigenic changes in gene expression. This study reports the first comparative multi-omics analysis of cancer-related changes in gene expression and DNA methylation in primary myofibroblasts derived from gastric and oesophageal tumours. In addition, we identify novel CAM-specific DNA methylation signatures, which are not observed in patient-matched adjacent tissue-derived myofibroblasts, or corresponding normal tissue-derived myofibroblasts. Analysis of correlated changes in DNA methylation and gene expression shows that different patterns of gene-specific DNA methylation have the potential to confer pro-tumourigenic changes in metabolism, cell signalling and differential responses to hypoxia. These molecular signatures provide new insights into potential mechanisms of stromal reprogramming in gastric and oesophageal cancer, while also providing a new resource to facilitate biomarker identification and future hypothesis-driven studies into mechanisms of stromal reprogramming and tumour progression in solid tumours.

## Introduction

Despite a recent decline in incidence (1), distal gastric cancer remains the third leading cause of cancer death worldwide (2). As such, there remains a need to develop a better understanding of the molecular processes that contribute to the development and metastasis of gastric tumours.

There is strong evidence that cancer-associated myofibroblasts (CAMs) exhibit tumour-promoting properties, which are not observed in myofibroblasts derived from non-cancerous tissue (3–7). Also, the presence of large numbers of CAMs within the tumour stroma is linked to poor prognosis (8,9) and resistance to therapy (10). Significantly, these tumour-promoting properties are retained in cultured gastric CAMs, when compared with patient-matched adjacent tissue myofibroblasts (ATMs) or normal tissue myofibroblasts (NTMs) (11,12), implying that some pro-tumourigenic properties may result from epigenetic reprogramming of CAMs within the tumour microenvironment. Interestingly, gastric and oesophageal CAMs both have distinct micro RNA signatures compared with corresponding populations of ATMs and NTMs (13). Also, gastric CAMs were reported to exhibit a global reduction in DNA methylation compared with patient-matched ATMs (14). However, previous studies were not performed at resolution, which allowed the mechanism, or functional consequences of CAM-specific DNA methylation changes to be assessed.

This study presents the first comparative genome-wide analysis of DNA methylation patterns at individual CpG resolution in primary gastric CAMs, patient-matched ATMs and unrelated gastric NTMs. Significantly, a subset of these molecular signatures was also observed in oesophageal adenocarcinoma-derived CAMs, suggesting that common mechanisms of stromal programming may operate in tumours derived from glandular cells in different tissues of the upper gastrointestinal tract. CAM-specific methylation patterns also provide potential stromal biomarkers, which may improve stratification and prognosis of both gastric and oesophageal tumours. To confirm the robust nature of cancer-related signatures identified in this study, comparative changes in the methylation status of a selection of genes with potential clinical relevance (*SMAD3*, *SPON2*, *FOXF1*, and *FENDRR*) were validated in an independent set of gastric CAMs, patient-matched ATMs and unrelated NTMs by pyrosequencing and quantitative PCR.

## Material and methods

### Generation and culture of human primary myofibroblasts

Human primary myofibroblasts derived from resected gastric and oesophageal adenocarcinomas (CAM) and adjacent tissue (ATM) were obtained from patients undergoing gastric or oesophageal cancer surgery (Supplementary Table 1, available at *Carcinogenesis* Online) as described previously (11,12). Normal gastric myofibroblasts (NTM) were generated from deceased transplant donors with normal morphology (Supplementary Table 2, available at *Carcinogenesis* Online) as reported previously (15). This work had been approved by the ethics committee of the University of Szeged, Hungary. Myofibroblasts were authenticated by quantitative PCR (qPCR) and immunocytochemistry as described previously (11,12). The analysis showed positive expression of α-smooth muscle actin and vimentin (myofibroblast and mesenchymal markers) and lack of desmin (pericyte marker) and cytokeratin (epithelial cell marker) expression. Cells were tested prior usage in a new study or at least every 6 months by immunocytochemistry to ensure that their phenotype is maintained. Primary myofibroblast cultures were maintained in Dulbecco’s modified Eagle’s medium supplemented with 10% foetal bovine serum, 1% penicillin–streptomycin, 1% antibiotic–antimycotic and 1% non-essential amino acid solution. Medium was replaced routinely every 48–60 h and cells were lysed at 80–90% confluence for DNA and RNA extraction. In all experiments, myofibroblast cells were not used beyond passage 12.

### Myofibroblast conditioned media preparation

To prepare CAM, ATM or NTM conditioned media (CM), 1.5 × 10^6^ myofibroblast cells were seeded in 75 cm^2^ tissue culture flasks and left to attach for 24 h. The next day, the cells were washed three times in 1× PBS to get rid of any serum-derived factors. Then growth media was replaced with 15 ml freshly prepared serum-free Dulbecco’s modified Eagle’s medium supplemented with 1% penicillin–streptomycin, 1% antibiotic–antimycotic and 1% non-essential amino acid solution and incubated for 24 h at 37°C in a humidified atmosphere with 5% CO_2_. The next day, CM was collected and centrifuged at 800*g* for 7 min to get rid of cell debris. The freshly prepared supernatants were immediately used for cancer cell migration and proliferation assays.

### Cancer cell migration assay

The effects of myofibroblast CM on gastric cancer cells migration were measured *in vitro* using transwell Boyden chamber assay (SLS; cat. no. 354578). Briefly, 1 × 10^4^ AGS cells in 500 μl serum-free Dulbecco’s modified Eagle’s medium were added to the 8 μm pore chambers. The lower chambers contained either 750 μl serum-free media or myofibroblast CM to serve as a chemoattractant. Cells were incubated at 37°C and allowed to migrate overnight. Cells migrating through the membrane were fixed and detected on the lower surface using Reastain Quick-Diff Kit (Reagena; cat. no. 102164). The total cells in 15 fields per well were counted, and the mean of at least three independent membranes per experiment was taken.

### Cancer cell proliferation assay

The effects of myofibroblast CM on gastric cancer cell proliferation were assessed by incorporation of EdU (16) and detected using the Click-iT EdU Alexa Fluor 488 Imaging Kit (Life Technologies; cat. no. C10337) according to the manufacturer’s instruction.

### DNA and RNA extraction

Genomic DNA was purified using a standard phenol/chloroform extraction method. Briefly, myofibroblast cells were lysed in lysis buffer (400 mM Tris-HCl, pH 8, 10 mM ethylenediaminetetraacetic acid, 1% sodium dodecyl sulfate, 150 mM NaCl). DNA quantity was assessed using PicoGreen fluorimetry (Life Technologies, cat. no. Q-33130). DNA samples were analysed by Molecular Genetic Services (Gen-Probe Life Sciences, Manchester) using Illumina Infinium HumanMethylation450k BeadChip arrays for analysis of DNA methylation. Total RNA was purified using miRNeasy Mini Kit (Qiagen, cat. no. 217004) and sample degradation and purity was assessed using the Agilent 2100 Bioanalyzer and RNA 6000 Nano Kit (Agilent Technologies, cat. no. 5067-1512). Samples were sent to Molecular Genetic Services (Gen-Probe Life Sciences, Manchester) for gene expression analysis using Illumina HumanHT-12 v4 arrays.

### Pyrosequencing analysis

DNA was extracted from seven patient-matched CAM and ATM samples and four unrelated NTM samples using Wizard SV Genomic DNA Purification Kits (Promega, cat. no. A2360). In each case, 1 μg of genomic DNA was treated with sodium bisulphite using the EZ DNA Methylation-Gold Kit (ZymoResearch, cat. no. D5005). A full list of assays, primer sequences and annealing temperatures is shown in Supplementary Table 3, available at *Carcinogenesis* Online. Pyrosequencing templates were prepared by PCR amplification using HotStarTaq Master Mix Kit (Qiagen, cat. no. 203603), 5 μM biotinylated primer, 5–10 μM non-biotinylated primer (corresponding to 1:1 or 1:2 ratio in Supplementary Table 3, available at *Carcinogenesis* Online), 5 mM dNTPs (Qiagen, cat. no. 201900) and 3 μl (~60 ng) bisulfite-treated DNA. The PCR thermal profile consisted of initial denaturation at 95°C for 5 min, followed by 40 cycles including 95°C for 30 s, annealing temperature (Supplementary Table 3, available at *Carcinogenesis* Online) for 30 s, 72°C for 30 s. A final extension step of 72°C for 10 min was also included. Purified biotinylated PCR products were made single-stranded to act as a template in a pyrosequencing reaction run. PCR products were bound to streptavidin-coated Sepharose beads (GE Healthcare, cat. no. 17-5113-01), before washing, and denaturising in 0.2 M NaOH. 0.5 µM. Pyrosequencing primers were annealed to the purified single-stranded PCR products, and pyrosequencing was carried out using the Pyromark 96ID System (Qiagen). The methylation index for the analysed genomic region was calculated as the mean value of mC/(mC + C) for all examined CpG sites in the interrogated genomic region.

### qPCR (TaqMan) expression analysis

TaqMan gene expression assays were used to quantify messenger RNA levels of target genes in stromal myofibroblasts. RNA samples were extracted from six patient-matched gastric CAM and ATM samples. Total RNA was purified using miRNeasy Mini Kit (Qiagen, cat. no. 217004) and QuantiTect Reverse Transcription Kits (Qiagen, cat. no. 205311) were used for complementary DNA synthesis and qPCR assays performed on the StepOne system (Applied Biosystems). Amplification mixture contained 7.5 μl 2× TaqMan Universal Master Mix II (Life Technologies, cat. no. 4440042), 0.75 μl 20× TaqMan probe and primers, 1.25 μl 10× ACTB, 2 μl complementary DNA and 3.5 μl ddH_2_O, giving a final volume of 15 μl. TaqMan assays were either designed using Oligo 7.0 software (Molecular Biology Insights) and synthesized by Eurofins MWG (Germany) or purchased as predesigned assays from Life Technologies (UK). A list of assays, nucleotide sequences and PCR product sizes is shown in Supplementary Table 4, available at *Carcinogenesis* Online. Amplification mixtures were processed using standard conditions (50°C for 2 min and 95°C for 10 min followed by 45–50 cycles at 95°C for 15 s and 60°C or 61°C for 1 min). β-Actin was used as the endogenous control. The comparative ΔΔCt method was used to compute relative levels of target gene expression, subtracting Ct values of the endogenous control (β-actin) before comparing values to a calibrator sample, where the calibrator sample = 1.0 and other samples were expressed as *n*-fold relative to the calibrator.

Methods for data processing and bioinformatics analysis are provided in Supplementary File 1, available at *Carcinogenesis* Online.

## Results

### Gastric CAMs retain a pro-tumourigenic phenotype *in vitro* and exhibit a global reduction in DNA methylation

Previous studies have shown that primary CAMs retain pro-tumourigenic properties following isolation and culture (17), including the ability to enhance cancer-cell migration and proliferation (11,12,18). This phenotype was confirmed for all primary gastric myofibroblast populations used in this study (Supplementary Figures 1 and 2, available at *Carcinogenesis* Online). Illumina 450k probes that passed stringent filtering criteria were used to compute mean β-values as an indication of the global DNA methylation status of gastric myofibroblasts. In agreement with reported trends (14), gastric CAMs used in this study all exhibited a global reduction in DNA methylation compared with patient-matched ATMs (Wilcoxon test, *P* < 2.2 × 10^–16^) (Supplementary Figure 3A, available at *Carcinogenesis* Online).

### Genome-wide DNA methylation profiling of gastric myofibroblasts purified from different tissue microenvironments

**Figure 1.**
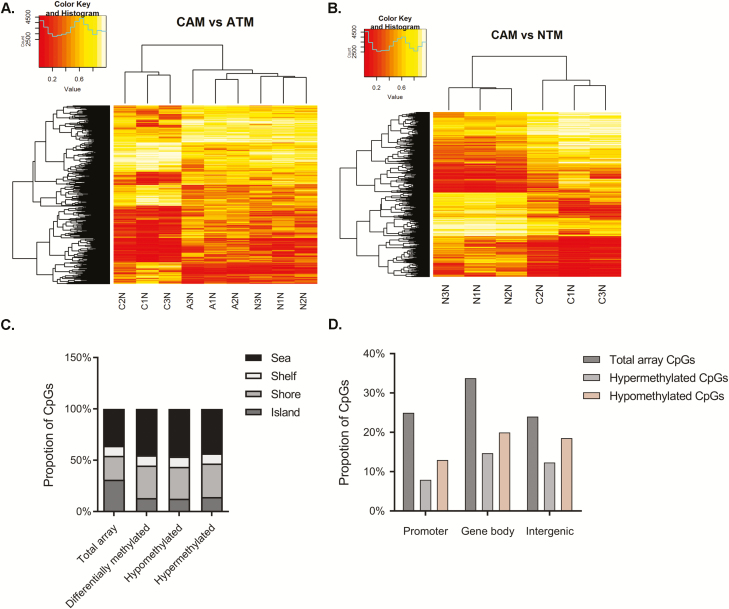
DNA methylation profiling of primary myofibroblasts purified from different tissue microenvironments. (**A)** Unsupervised clustering of 5688 CpG loci with marked differential methylation in CAMs and patient-matched ATMs with projection including related values observed in NTMs. (**B)** Unsupervised clustering of 8104 CpG loci with marked differential methylation in CAMs and unrelated NTMs. Heatmaps represent differentially methylated CpG loci identified in respective comparisons |Δβ| > 0.2, *P* value < 0.05. (**C** and **D**) Distribution of differentially methylated CpG loci identified in CAMs versus ATMs (C) in CpG islands, shores, shelves and sea regions or (D) relative to RefSeq gene promoters, gene bodies and intergenic regions.

**Figure 2.**
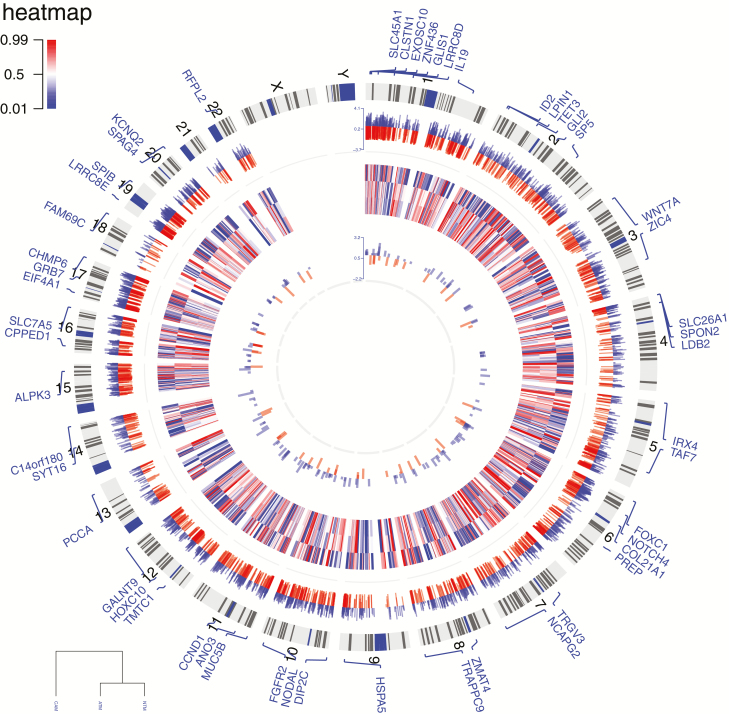
Circular plot of genome-wide DNA methylation changes in stromal myofibroblasts. The outer ring represents human ideograms. The first track shows differentially methylated CpG sites between gastric CAMs and patient-matched ATMs. The heatmap represents CpG loci that distinguish CAMs from non-tumour derived myofibroblasts (ATMs and NTMs). These loci may serve as proxies for the identification of gastric CAMs. The innermost track represents conserved CAM susceptible loci identified in both gastric and oesophageal CAMs compared to patient-matched ATMs; red—hypermethylated loci in CAMs, blue—hypomethylated loci in CAMs; |Δβ| > 0.2, *P* value < 0.05.

To provide new insight into epigenetic changes that may be linked to the tumour-promoting properties of gastric CAMs, a comparative genome-wide DNA methylation analysis was performed on sets of gastric CAMs, patient-matched ATMs and unrelated NTMs, using the Illumina Infinium HumanMethylation450k BeadChip. This analysis identified numerous CpG sites that show consistent differences in DNA methylation in CAMs compared with either ATMs or NTMs. In total, 5688 differentially methylated CpG sites were identified in CAMs compared with ATMs, including 3404 hypomethylated and 2284 hypermethylated CpG sites. These loci were more frequently located in CpG shores than CpG islands. Comparison of the overall distribution of differentially methylated loci relative to RefSeq genes showed that hypomethylated CpG loci were over-represented in promoters, gene bodies and intergenic regions (Figures 1 and 2, outer track). In the CAM versus NTM comparison, a total of 8104 differentially methylated CpG loci were identified, including 4147 and 3957 loci that were respectively hypo- or hypermethylated in CAMs. These genome-wide and gene-specific methylation patterns provide important signatures that facilitate differentiation between tumour-derived myofibroblasts (CAMs) and non-tumour-derived myofibroblasts (ATMs or NTMs). Equally, identification of CAM-specific DNA methylation changes may aid biomarker identification for improved diagnosis/prognosis or tumour/patient stratification. Therefore, a comparative analysis was performed to identify CpG loci that distinguish CAMs from non-tumour-derived myofibroblasts, following CAM versus ATM and CAM versus NTM differential methylation analysis. The resulting overlap of 2006 CpG loci from these comparisons was then selected. Multiple CpG loci were found to be hypomethylated in CAMs but hypermethylated in both ATMs and NTMs and *vice versa* (Supplementary Figure 4, available at *Carcinogenesis* Online; Figure 2, heatmap). As these genomic regions show distinct DNA methylation patterns in myofibroblast populations, it is possible that they facilitate distinction between different types of gastric myofibroblasts and different stages, or degrees of tumour reprogramming.

### Technical validation of novel cancer-related changes in DNA methylation

Pyrosequencing assays were performed to validate the methylation level of CpG sites identified in gastric CAMs by Illumina 450k arrays. Supplementary Figure 5, available at *Carcinogenesis* Online, shows correlations between β-values and methylation index assessed by pyrosequencing in DNA samples that were used in the initial array experiments. Correlations between the two types of DNA methylation assays for 12 CpG loci interrogated were high (*R*^2^ = 0.8177–0.9921, *P* value = 7.1 × 10^–3^–1.41 × 10^–7^), thus increasing confidence in the reliability of comparative differential DNA methylation trends identified in this study.

### *In silico* molecular enrichment analysis

To investigate the potential functional consequences of differentially methylated CpG loci identified in gastric CAMs, Gene Ontology (GO) enrichment and Ingenuity Pathway Analysis (IPA) were performed on subsets of genes with associated changes in methylation status. GO enrichment analysis revealed that aberrant DNA methylation has the potential to affect biological processes that are directly associated with tumour growth and progression, including the regulation of cell development and differentiation, cell adhesion, chemotaxis, transmembrane transport, regulation of cholesterol biosynthesis and extracellular matrix organization (Supplementary File 2, available at *Carcinogenesis* Online). Equally, IPA identified processes involving aberrantly methylated genes, including gene expression, cellular movement, cell growth and proliferation, cellular development and morphology, cell signalling, energy production and lipid metabolism. Interestingly, transforming growth factor (TGF-β) signalling was identified as one of the most over-represented pathways that may be affected by cancer-imposed changes in CAM DNA methylation (Supplementary File 3, available at *Carcinogenesis* Online).

### Gene expression profiling of gastric myofibroblast purified from different tissue microenvironments

A comparative genome-wide gene expression analysis was performed on populations of gastric CAMs, ATMs and NTMs using the Illumina HumanHT-12v4 Expression BeadChip arrays. The analysis revealed 13 381 consistently expressed genes.

**Figure 3.**
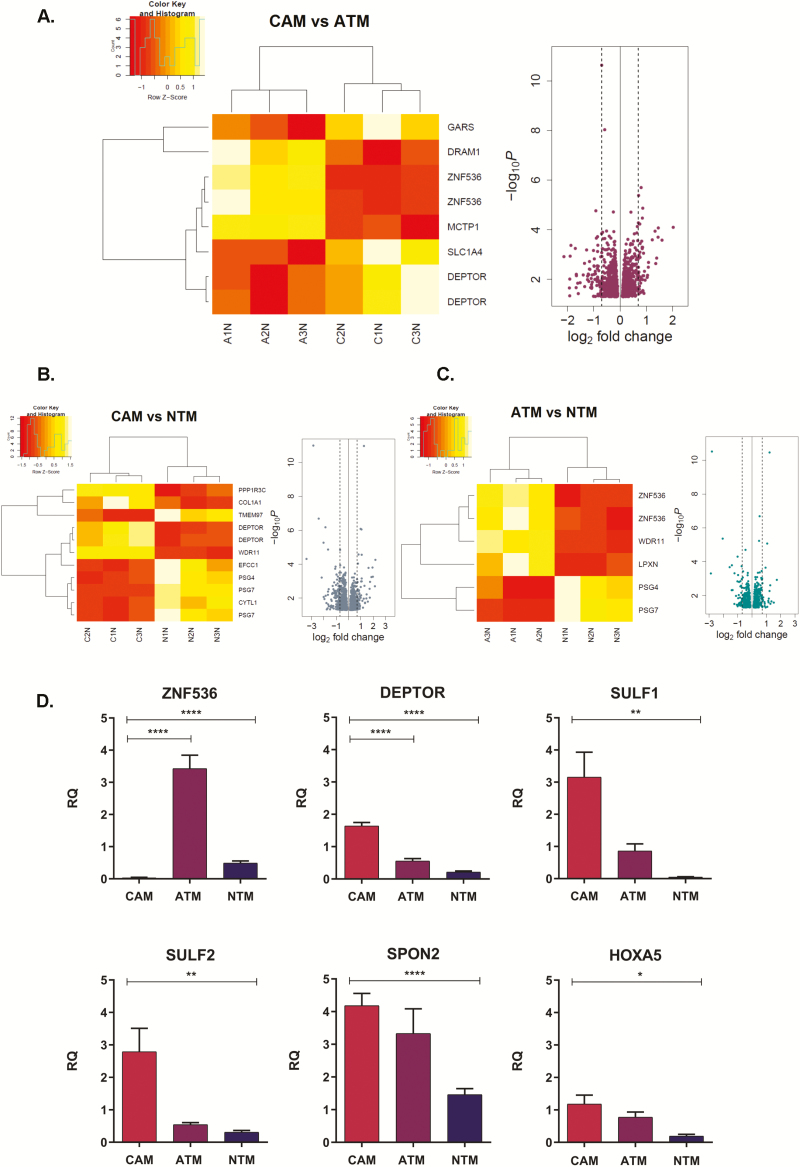
Differential gene expression signatures in gastric myofibroblasts purified from different tissue microenvironments. (**A**) CAM versus ATM. (**B**). CAM versus NTM. (**C)** ATM versus NTM. Heatmaps represent differentially expressed genes in respective comparisons *FDR P value < 0.05;* volcano plots represent differentially expressed genes *P value < 0.05;* dashed lines 1.6-fold change. (**D**) Quantitative PCR validations of genes identified as differentially expressed in CAM versus ATM and CAM versus NTM comparisons. Each TaqMan assay was performed in triplicates for CAM (*n* = 3), ATM (*n* = 3) and NTM (*n* = 3) samples. The comparative ΔΔCt method was used and samples were normalized to calibrator. Error bars represent standard error of mean; CAM versus ATM *t* test ****P < 0.0001; CAM versus NTM *t test* ****P<0.0001, **P<0.01, *P < 0.05.

Pairwise differential gene expression analyses performed between CAM versus ATM, CAM versus NTM and ATM versus NTM identified: 1215 genes (574 upregulated and 641 downregulated in CAMs) that were differentially expressed between CAMs and patient-matched ATMs; 987 genes that were differentially expressed between CAMs and unrelated NTMs (508 upregulated and 479 downregulated in CAMs) and 713 genes that were differentially regulated in ATMs compared with NTMs (407 upregulated and 306 downregulated in ATMs; Figure 3A–C).

### Technical validation of CAM-specific changes in gene expression

To validate the results from the differential gene expression analysis, candidate genes from CAM versus ATM and CAM versus NTM comparisons were selected and analysed by TaqMan qPCR (Figure 3D). Triplicate reactions were conducted on RNA samples that were used in the initial array experiment, and data were analysed using the comparative ΔΔCt method. Quantitative analysis of candidate genes confirmed the expression patterns observed by Illumina HT-12 array analysis, thus increasing confidence in the identified comparative differential gene expression trends.

### *In silico* enrichment analysis

To assess processes that may be altered by induced changes in gene expression in gastric CAMs, a combination of GO and Gene Set Enrichment analysis was performed (Supplementary Figure 6, Files 4 and 5, available at *Carcinogenesis* Online). Significantly, 20% genes differentially expressed between CAMs and patient-matched ATMs are known components of secretory exosomes (19). Although these data provide a new resource to drive future studies into the mechanisms by which stromal myofibroblasts promote tumour growth, it is important to establish which of the differential gene expression signatures may result from CAM-specific changes in DNA methylation.

### Integration of CAM-specific DNA methylation and gene expression profiles

Although the relationship between DNA methylation and gene expression is complex, in some cases, the degree of promoter methylation is inversely correlated with gene expression, whereas methylation in gene bodies often shows a positive correlation with gene expression (20,21). This study identified a subset of 419 genes that show CAM-specific changes in both DNA methylation and gene expression; 230 of these genes are differentially methylated upstream of their annotated transcriptional start site, whereas 254 genes show altered DNA methylation patterns within gene bodies, with 65 genes exhibiting changes in both regions (Supplementary Figure 7, available at *Carcinogenesis* Online).

Of the 124 genes with coordinated changes in promoter methylation and gene expression, 55 were hypermethylated and downregulated, whereas 69 were hypomethylated and upregulated in comparison with corresponding ATMs (Supplementary Figure 7B, available at *Carcinogenesis* Online). These data suggest that hypo- and hypermethylated loci encode functionally distinct genes. In particular, genes that were hypermethylated and downregulated in CAMs were over-represented in highly relevant functional classes, including gastrointestinal adenocarcinoma, gastrointestinal tract cancer, gastro-oesophageal carcinoma and gastric cancer. In contrast, CAM-specific genes that were hypomethylated and upregulated were not associated with these processes. However, they did show enrichment in processes linked to extracellular vesicular exosomes, transport of amino acids and secretion of molecules (Supplementary Table 5, available at *Carcinogenesis* Online).

With respect to the 152 genes that showed correlated changes in gene body methylation and gene expression (Supplementary Figure 7B, available at *Carcinogenesis* Online), 79 genes that were hypomethylated and repressed were primarily associated with invasion, proliferation, transformation, transport of molecule and migration, whereas 73 genes that were hypermethylated and induced show greater association with metabolic processes such as metabolism of amino acids and metabolism of heparin sulphate proteoglycans (Supplementary Table 6, available at *Carcinogenesis* Online).

### Verification of CAM-specific DNA methylation and gene expression signatures

To investigate the impact of cancer-induced changes in DNA methylation on gene expression, pyrosequencing methylation and qPCR expression analyses were performed on an additional set of independent gastric CAMs, patient-matched ATMs and unrelated gastric NTMs, which were not included in initial genome-wide profiling studies. Targeted pyrosequencing assays were performed on genomic regions associated with the regulation of a subset of candidate genes, including *SMAD3*, *SPON2*, *FOXF1* and *FENDRR*, all of which have been implicated previously in cancer and tumour-stroma communication (19,22–24) and were shown to be differentially methylated in this study.

### Promoter hypermethylation represses *SMAD3* expression in gastric CAMs

**Figure 4.**
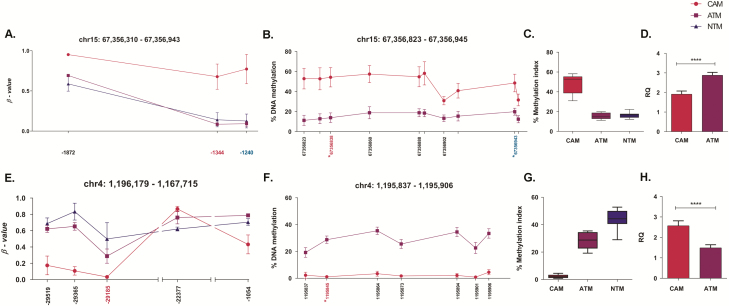
Changes in DNA methylation in the SMAD3 and SPON2 promoter regions regulates gene expression in gastric CAMs and ATMs. (**A**) Differentially methylated CpG loci identified by Illumina 450k array in the SMAD3 promoter region. Mean β values (*n* = 3) for probes identified as differentially methylated in CAM versus ATM and CAM versus NTM comparisons. The *x*-axis indicates the distance of Illumina 450k probes from SMAD3 transcription start site. Positions highlighted in magenta (–1344) and blue (–1240) are within the genomic region examined by corresponding pyrosequencing assays. (**B**) Pyrosequencing analysis of the SMAD3 promoter region was performed on patient-matched CAM (*n* = 7) and ATM (*n* = 7) samples. Methylation means for 10 individual CpG sites in the SMAD3 promoter region are plotted. The *x*-axis indicates the chromosomal position of examined CpG sites. Positions marked with * correspond to the Illumina 450k probes. (**C**) The overall methylation level of the SMAD3 promoter region interrogated by pyrosequencing analysis. Boxplots represent methylation distribution and mean for 10 CpG sites in CAM (*n* = 7), ATM (*n* = 7) and NTM (*n* = 4) samples. (**D**) Quantitative PCR analysis of SMAD3 gene expression in CAM (*n* = 6) and ATM (*n* = 6) samples; t test ****P < 0.0001. Error bars represent SEM. DNA methylation in the SPON2 promoter region correlates with SPON2 gene expression in gastric CAMs and ATMs. (**E**) Differentially methylated CpG sites identified by Illumina 450k array in the SPON2 promoter region. Mean β values (*n* = 3) for probes found to be differentially methylated in CAM versus ATM and CAM versus NTM comparisons. The *x*-axis indicates the distance of Illumina 450k probes from the SPON2 transcription start site. Position highlighted in magenta (–29185) is within the genomic region examined by pyrosequencing. (**F**) Pyrosequencing analysis of the SPON2 promoter region in patient-matched CAM (*n* = 7) and ATM (*n* = 7) samples. Methylation means for seven individual CpG sites in the interrogated promoter region are plotted. The *x*-axis indicates the chromosomal position of examined CpG sites. Position marked with * corresponds to the Illumina 450k probe highlighted in magenta (–29185) in Panel E. (**G)** The overall methylation level of the SPON2 promoter region interrogated by pyrosequencing. Boxplots represent methylation distribution and mean for seven CpG sites in CAM (*n* = 7), ATM (*n* = 7) and NTM (*n* = 4) samples. (**H)** Quantitative PCR analysis of SPON2 gene expression in CAM (*n* = 6) and ATM (*n* = 6) samples; t test ****P < 0.0001. Error bars represent standard error of mean.

Two pyrosequencing assays were designed for *SMAD3* covering a 224 bp region spanning 10 CpG sites, including 2 CpGs identified in Illumina 450k arrays. These assays confirmed that the *SMAD3* promoter is hypermethylated in CAMs (Figure 4A and B). Notably, DNA methylation levels in the *SMAD3* promoter region were found to be very similar in ATMs and NTMs (Figure 4C), whereas qPCR expression analysis confirmed that SMAD3 is significantly downregulated in CAMs (Figure 4D). Therefore, this genomic region may provide a proxy/biomarker for gastric CAM identification. Interestingly, *SMAD3* hypermethylation in tumours is associated with poorer overall survival (Supplementary Figure 9, available at *Carcinogenesis* Online). Collectively, these data provide a strong indication that *SMAD3* expression may be repressed by cancer-induced reprogramming, resulting in *SMAD3* promoter hypermethylation in gastric CAMs.

### Promoter hypomethylation induces *SPON2* expression in gastric CAMs

To investigate cancer-induced changes in *SPON2* expression, a pyrosequencing assay was designed for *SPON2* covering 117 bp spanning 7 CpG sites, including 1 CpG site identified by the Illumina 450k array (Figure 4E and F). The pyrosequencing assay confirmed *SPON2* promoter hypomethylation in CAMs, whereas qPCR assay confirmed that *SPON2* expression is upregulated in CAMs. Interestingly, pyrosequencing data show that the extent of *SPON2* promoter DNA methylation gradually changes in gastric stromal myofibroblasts, with low levels in CAMs, intermediate levels in patient-matched ATMs and high levels in NTMs (Figure 4G). Significantly, these trends show a good negative correlation with *SPON2* expression patterns (Figures 3D and 4H) and protein levels (Supplementary Figure 10, available at *Carcinogenesis* Online) observed in secretome of these cells (data not shown). Taken together, these data provide strong evidence that *SPON2* expression may be regulated by cancer-induced differential promoter DNA methylation in gastric CAMs, ATMs and NTMs.

### DNA hypermethylation represses the expression of *FOXF1* and *FENDRR* in gastric CAMs

**Figure 5.**
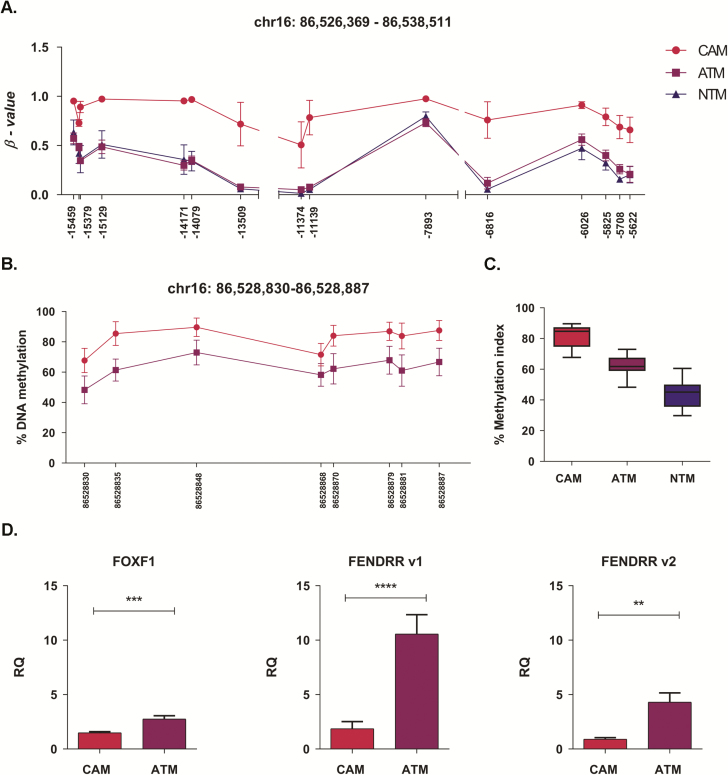
DNA methylation pattern in the genomic region associated with regulation of *FOXF1* and *FENDRR* expression in gastric CAMs and ATMs. (**A**) Differentially methylated CpG sites identified by Illumina 450k array in the region downstream of the FOXF1 transcription start site. Mean β values (*n* = 3) for probes identified as differentially methylated in CAM versus ATM and CAM versus NTM comparisons are plotted. The *x*-axis indicates the distance of Illumina 450k probes to *FOXF1* transcription start site. (**B**) Pyrosequencing analysis of the *FOXF1* promoter region in patient-matched CAM (*n* = 7) and ATM (*n* = 7) samples. Methylation means for eight individual CpG sites in the interrogated promoter region are plotted. The *x*-axis indicates chromosomal position of examined CpG sites. (**C**) The overall methylation level of the FOXF1 promoter region interrogated by pyrosequencing assay. Boxplots represent methylation distribution and mean for eight CpG sites in CAM (*n* = 7), ATM (*n* = 7) and NTM (*n* = 4) samples. (**D**) Quantitative PCR analysis of *FOXF1* and *FENDRR* gene expression in CAM and ATM samples; *t* test *FOXF1* (*n* = 5) ***P = 0.0008; *FENDRR*, v1 (splice variant 1; *n* = 4) ****P < 0.0001; *FENDRR*, v2 (splice variant 2; *n* = 4) **P = 0.0028. Error bars represent standard error of mean.

**Figure 6.**
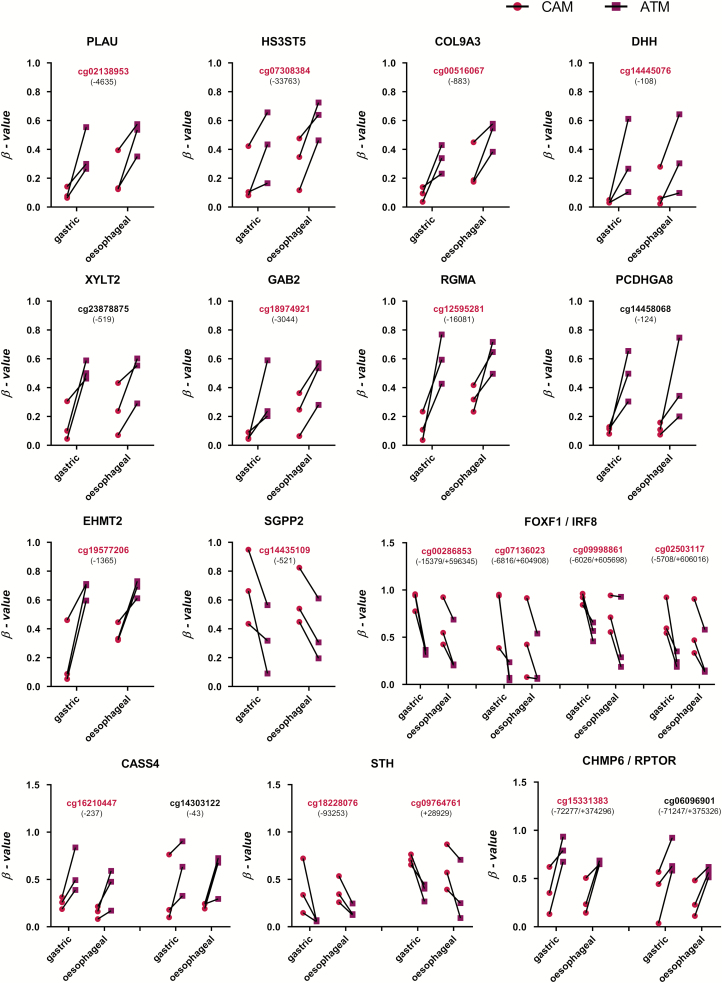
Representative conserved DNA methylation patterns in gastric and oesophageal patient-matched CAM and ATM samples. Probes highlighted in magenta are also identified as proxies for gastric CAMs. Numbers in brackets indicate the distance to transcription start site (TSS) of a given gene (indicated at the top of each plot); magenta—CAMs, purple—ATMs; |Δβ| > 0.2, *P* value < 0.05.

A region on chromosome 16 spanning 526 426 bp was identified as one of the largest differentially methylated regions in gastric CAM versus ATM and CAM versus NTM comparisons (Figure 5A). Notably, a smaller part of this region (chr16: 86 528 753–86 538 425) spanning 9673 bp was also identified as differentially methylated in oesophageal CAM versus ATM comparison (Supplementary Figure 11, available at *Carcinogenesis* Online; Figure 6). Differential DNA methylation within this region may regulate the expression of *FOXF1* and several long non-coding RNAs, including *FENDRR*.

To assess whether *FOXF1* and *FENDRR* expression is regulated by DNA methylation in gastric CAMs, a pyrosequencing assay was designed to investigate the *FOXF1* promoter region, which also overlaps with the *FENDRR*. The assay covers 104 bp spanning 8 CpG sites (Figure 5B). This analysis confirms that the *FOXF1* promoter region is consistently hypermethylated in CAMs when compared with either ATMs or NTMs. Notably, the *FOXF1* promoter shows a gradual change in DNA methylation levels CAMs > ATMs > NTMs (Figure 5C). Interestingly, this genomic region is commonly hypermethylated in gastric cancer (Supplementary Figure 12, available at *Carcinogenesis* Online), suggesting that CAMs might acquire some of the cancer-like DNA methylation patterns. In addition, qPCR analysis shows that *FOXF1* and *FENDRR* expression are both downregulated in CAMs compared with ATMs (Figure 5D). Collectively, these data provide a strong indication that *FOXF1* and *FENDRR* expression may be reprogrammed by DNA methylation within this region in both gastric CAMs and ATMs.

Data from these extended pyrosequencing and qPCR studies provide further evidence that CAM-specific promoter DNA methylation patterns may regulate the expression of associated genes. In addition, pyrosequencing analysis confirmed the existence of DNA methylation changes within border genomic regions, spanning several neighbouring CpG sites, between gastric CAMs and non-tumour-derived myofibroblasts (ATMs and NTMs).

### Comparison of DNA methylation patterns in CAMs derived from different tumour types

Although the primary aim of this study was to identify genome-wide DNA methylation patterns in CAMs derived from gastric tumours, it was pertinent to question if CAMs that are reprogrammed in different adenocarcinomas from the upper gastrointestinal tract show common changes in DNA methylation. To address this question, genome-wide DNA methylation profiles of primary patient-matched CAMs and ATMs, derived from oesophageal tumours, were compared with signatures observed in gastric CAMs and ATMs.

The genome-wide analysis identified widespread DNA methylation alterations and confirmed that oesophageal CAMs also exhibit a global loss of DNA methylation compared with corresponding patient-matched ATMs (Supplementary Figure 3B-C, available at *Carcinogenesis* Online). To identify common differentially methylated genes in gastric and oesophageal CAMs, differentially methylated CpG loci identified in both sets of CAMs were assigned to genes. Differentially methylated CpG loci from gastric CAMs were assigned to 5918 genes, whereas differentially methylated oesophageal CAM CpG loci were assigned to 4105 genes. Comparison of these gene lists identified 2223 common genes, which evidence of cancer-induced changes in DNA methylation in both gastric and oesophageal CAMs (Supplementary Figure 8, available at *Carcinogenesis* Online). Notably, comparison analysis of differentially methylated CpG sites found 230 CpGs that are differentially methylated in both gastric and oesophageal CAMs when compared with corresponding patient-matched ATMs (Figure 2 innermost track; Figure 6). These conserved differentially methylated loci are distributed throughout the genome (Figure 2 innermost track). Further analysis of these loci shows that 65.22% are associated with two genes and 33.5% are associated with only one gene.

To investigate how DNA methylation changes in gastric and oesophageal CAMs may affect common pathways and processes, 2223 common differentially methylated genes were subjected to IPA and ConsensusPathDB (25) over-representation analysis (Enriched KEGG and Reactome pathways are reported in Supplementary File 6, available at *Carcinogenesis* Online). IPA showed that these commonly differentially methylated genes are involved in digestive organ tumour (*P =* 1.67 × 10^–17^), expression of RNA (*P =* 3.46 × 10^–12^), transcription (*P =* 7.36 × 10^–12^), gastro-oesophageal cancer (*P =* 7.94 × 10^–11^), tumourigenesis of the tissue (*P =* 3.13 × 10^–11^), cell movement (*P =* 1.83 × 10^–10^), migration of cells (*P =* 2.18 × 10^–9^), proliferation of cells (*P =* 1.28 × 10^–8^), invasion of cells (*P =* 3.43 × 10^–6^), generation of fibroblasts (*P =* 1.69 × 10^–5^) and growth of tumour (*P =* 4.97 × 10^–5^).

In addition, the results from the gastric and oesophageal GO enrichment analysis of differentially methylated CpG loci (Supplementary Files 2 and 7, available at *Carcinogenesis* Online) were compared to get further insight into common biological processes affected by DNA methylation changes; 32 unifying GO biological processes were identified (Supplementary Figure 8, available at *Carcinogenesis* Online). Enriched biological processes linked to cancer-related changes in DNA methylation in gastric and oesophageal CAMs include cell adhesion, cell differentiation and developmental processes, signalling, regulation of signal transduction and guanosine triphosphatase activity. As gastric stromal myofibroblasts have a neuroendocrine-like phenotype, which is associated with advanced cancer (15), it is interesting to note that synaptic transmission and regulation of calcium transport were one of the significantly enriched biological processes targeted by DNA methylation changes in both gastric and oesophageal CAMs (Supplementary File 2 and 7, available at *Carcinogenesis* Online). These conserved signatures represent an additional resource to inform future hypothesis-driven studies into conserved mechanisms or functional consequences of tumour-induced stromal reprogramming.

## Discussion

As the tumour microenvironment plays an important role in cancer progression, increasing efforts are being made to understand the molecular processes that drive pro-tumourigenic changes in stromal myofibroblasts.

Although differential patterns of DNA methylation are well documented in cancer cells (26–28), there is relatively little information relating to induced epigenetic changes in stromal myofibroblasts derived from different tumours. Consequently, it is not yet clear to what extent common mechanisms of stromal reprogramming operate in different solid tumours. In this study, an integrated multi-omics approach was used to provide the first evidence that myofibroblasts derived from the site of gastric and oesophageal adenocarcinomas (CAMs) are epigenetically reprogrammed to have distinct DNA methylation signatures, compared with non-tumour-derived patient-matched myofibroblasts (ATMs) or corresponding tissue-matched non-tumour-associated myofibroblasts (NTMs).

Previous knowledge of genome-wide epigenetic changes within tumour stroma was largely based on the analysis of epithelial, myoepithelial and stromal fibroblasts, derived from either normal breast tissue or in situ and invasive breast carcinomas (29). These studies show that distinct epigenetic profiles were observed in tumour-associated fibroblasts, revealing both stage and cell-type-specific variations (29). However, the link between imposed epigenetic changes and the molecular processes that contribute to reciprocal interactions between cancer and stromal myofibroblasts remains incomplete. CAMs derived from gastric (14) or non-small-cell lung cancer (30) were found to exhibit reduced global DNA methylation, accompanied by a selective gain in focal DNA methylation. Significantly, all primary gastric and oesophageal CAMs used in this study also exhibited reduced global DNA methylation levels in comparison with patient-matched ATMs. Given the emerging consistency between CAMs derived from different tumours, a subtle yet significant reduction in global DNA methylation may be a useful common indicator of a functional transition from NTM or ATM status, to a cancer-promoting CAM phenotype.

Despite growing evidence that functional differences in myofibroblast populations are linked to conserved changes in DNA methylation, previous studies did not investigate CAM-specific DNA methylation profiles at single CpG resolution (14,29). As such, the molecular mechanisms and functional consequences of cancer-induced epigenetic programming remained unclear. In this context, this study provides new insight into CAM-specific changes, identifying CpG loci with altered DNA methylation in both gastric and oesophageal CAMs compared with patient-matched ATMs or unrelated NTMs. Interestingly, unsupervised clustering, based on genome-wide DNA methylation profiles, shows that ATMs are more similar to NTMs then to corresponding patient-matched CAMs. Also, loci that were consistently hypomethylated in CAMs were commonly hypermethylated in both ATMs and NTMs. Thereby, providing evidence that CAMs are uniquely reprogrammed to have common gene-/loci-specific patterns of methylation. Therefore, novel conserved signatures may provide further insight into the molecular mechanism and functional consequences of cancer-induced epigenetic reprogramming. Also, CAM-specific patterns of DNA methylation may facilitate the identification of proxy markers of regional stromal conversion.

Interestingly, altered patterns of promoter methylation observed in a number of genes (e.g. *ZMIZ1*, *EYA4*, *SLC22A18AS*, *WIPF1*, *FAM49A*, *RUNX3* and *ESRRG*) in both gastric and oesophageal CAMs were also reported previously in CAMs derived from lung tumours (30), indicating that common mechanisms of epigenetic reprogramming may contribute to the aberrant expression of these genes in CAMs derived from different tumours.

Functional enrichment analysis of data from this study shows that CAM-specific changes in DNA methylation have the potential to affect processes related to tumour development, including digestive organ tumours, tumourogenesis, cell movement, cell migration and proliferation, generation of fibroblasts, growth of tumours and cell adhesion. Loci showing CAM-specific changes in methylation were also found to encode genes associated with Hedgehog, Wnt and Notch signalling pathways, several of which are differentially expressed in both gastric and oesophageal cancer (31–33). Although deregulation of these pathways has been linked to tumour development, the spectrum of cell types that contribute to the observed signals *in vivo* remains unclear. Loci-encoding genes involved in glycosaminoglycan biosynthesis and metabolism, an important component of extracellular matrix involved in cell signalling, cell function and cancer progression (34), were also found to be differentially methylated in isolated gastric and oesophageal CAMs. In terms of reprogramming energy production in CAMs, it is interesting to note that CAM-specific DNA methylation signatures have the potential to affect fatty acid, triacylglycerol and ketone body metabolism, providing the first evidence that epigenetic reprogramming may contribute to a reverse Warburg phenotype in gastric and oesophageal CAMs. Interestingly, this phenotype would also be associated with a concomitant reduction in mitochondrial activity, which may, in turn, contribute to a global reduction in DNA methylation (35–38). In terms of paracrine communication within the tumour microenvironment, the previous study in gastric has shown that myofibroblasts have neuroendocrine-like properties, which are lost in advanced stages of cancer (15). Interestingly, both synaptic transmission and regulation of Ca^2+^ transport were identified as biological processes affected by CAM-specific changes in DNA methylation.

Further insight into the functional implications of CAM-specific changes in DNA methylation was provided by parallel genome-wide gene expression profiling of gastric CAM, ATM and NTM isolates. Previous studies in breast (39–42), non-small-cell lung cancer (43), colon (44) and oral squamous cell carcinoma (45) reported CAM-specific changes in gene expression, compared with patient-matched ATMs or tissue-matched NTMs. In each case, CAM-specific gene expression profile may provide proxy marker for diagnosis or prognostic predictions. As in the case of oral squamous cell carcinoma, where gene expression studies identified two distinct CAM subtypes with differential tumour-promoting abilities (46) and showed that CAM expression profiles reflect the stage of tumour progression (45).

To assess the validity of correlated DNA methylation/gene expression signatures, a subsequent series of targeted pyrosequencing and qPCR studies were performed on additional set of gastric CAMs, ATMs and NTMs. This analysis confirmed initial CAM-specific patterns of DNA methylation/gene expression, while also revealing equivalent changes in DNA methylation in associated border genomic regions. These verification studies increase confidence in data derived from genome-wide studies, while also providing insight into the mechanisms by which imposed changes in CAM DNA methylation contribute to tumour progression.

As promoter hypermethylation of *SMAD3* correlates with reduced gene expression in gastric CAMs, it is likely that these imposed changes may perturb TGF-β signalling responses. Interestingly, our data show that simultaneous promoter hypermethylation and gene body hypomethylation result in the transcriptional repression of *TGFBR2* (type II TGF-β receptor). As selective ablation of *TGFBR2* in mouse stromal fibroblasts induced neoplastic lesions and stromal expansion (47), it is possible that combined attenuation to TGF-β signalling in CAMs may play a key role in the development of gastric and oesophageal tumours. Notably, *SMAD3* was also found to be downregulated in oesophageal CAMs and Vizoso *et al.* (30) also reported promoter hypermethylation-associated SMAD3 silencing in CAMs derived from lung tumours. In a related analysis of a panel of wound-related ECM genes, *COL1A1*, *EDA-FN*, *LOX* and *SPARC* were all found to be upregulated in CAMs compared with patient-matched control fibroblasts (30). Significantly, we also found that *COL1A1*, *SPARC* and *LOX* were upregulated in gastric CAMs compared with patient-matched ATMs. Collectively, these observations suggest that promoter silencing of *SMAD3* expression may contribute to CAM aberrant phenotype and underlie tumour-promoting properties of CAMs derived from different tissues.

Extended verification studies confirmed a recurrent CAM-specific signature in the genomic region encoding the FOX1 adjacent non-coding developmental regulatory RNA (*FENDRR*) and *FOXF1* genes. *FENDRR* is a long non-coding RNA transcribed bidirectionally on the opposite strand to the *FOXF1* gene, which encodes a transcription factor involved in embryonic development and mesenchymal–epithelial interaction (22). Also, there is evidence that *FOXF1* acts as a tumour suppressor, as it is inactivated by DNA methylation in breast cancer (48) and its expression is reduced in prostate cancer (49). This study shows that cancer-imposed changes in DNA hypermethylation may lead to reduced expression of *FENDRR* and *FOXF1* in gastric CAMs. Significantly, Xu *et al.* (23) showed that *FENDRR* regulates gastric cancer metastasis and is downregulated in gastric cancer cells relative to cells derived from normal gastric epithelial. Low levels of *FENDRR* were also found to correlate with poor patient prognosis and more aggressive tumour characteristics including greater invasion depth, higher tumour stage and lymphatic metastasis (23). *FOXF1* has also been shown to contribute to the tumour-promoting properties of lung cancer-associated fibroblasts, including the production of hepatocyte growth factor and fibroblast growth factor-2, both of which promote tumour growth (50).

In conclusion, findings presented in this study provide new insight into the imposed molecular changes that contribute to epigenetic and functional reprogramming of gastric CAMs thereby improving our understanding of the complex range of reciprocal interactions that occur between developing tumours and the stromal microenvironment. Further studies are needed to establish the extent to which identified trends are also observed at protein level. As DNA methylation patterns are more robust and long-lasting than messenger RNA or protein signatures, the signatures that differentiate CAM, ATM and NTM methylation profiles may also provide a useful resource to identify new markers to improve tumour stratification and the ability to define not just tumour boundaries but also the surrounding region of stromal reprogramming, which may be an important factor in defining optimal resection margins.

## Funding

Cancer Research UK non-clinical training award (C35628/A12779). Funding to pay the Open Access publication charges for this article was provided by the University of Liverpool.

## 

Conflict of interest: The authors declare no conflict of interest.

## Supplementary Material

bgz001_suppl_Supplementary_File_S1

bgz001_suppl_Supplementary_File_S2

bgz001_suppl_Supplementary_File_S3

bgz001_suppl_Supplementary_File_S4

bgz001_suppl_Supplementary_File_S5

bgz001_suppl_Supplementary_File_S6

bgz001_suppl_Supplementary_File_S7

bgz001_suppl_Supplementary_Figures

## Abbreviations

ATM: adjacent tissue-derived myofibroblast
CAM: cancer-associated myofibroblast
CM: conditioned media
GO: Gene Ontology
IPA: Ingenuity Pathway Analysis
NTM: normal tissue-derived myofibroblast
qPCR: quantitative PCR
TGF: transforming growth factor
