# Supplementary material for: Integrated omics profiling reveals novel patterns of epigenetic programming in cancer-associated myofibroblasts
Source: Carcinogenesis. 2019 Jan 8;40(4):500–12. doi: 10.1093/carcin/bgz001 (PMC6556705; doi:10.1093/carcin/bgz001)
Supplement: bgz001_suppl_Supplementary_File_S1 [file bgz001_suppl_supplementary_file_s1.docx]

Supplementary Methods

**Data processing and informatic analysis**

**Illumina Infinium 450k data processing and analysis**

To assess genome-wide DNA methylation changes in stromal myofibroblasts, Illumina Infinium HumanMethylation450 BeadChip arrays were performed on a collection of primary gastric and oesophageal patient-matched CAM and ATM (n=3) and unrelated gastric NTM samples (n=3). The Illumina 450k array interrogates over 480,000 CpG loci distributed across the genome at single base resolution, providing coverage of 98.9% UCSC RefGenes [1]. Raw DNA methylation data were processed and analysed using Bioconductor package RnBeads version 0.99.17 [2]. Briefly, raw data files (.IDAT) were imported directly into RnBeads. The comparison of non-CpG SNP probes present on the array (n=65) confirmed that CAMs and ATMs were sourced from the same patient as expected. The methylation level (β-value) for the interrogated CpG loci was computed by calculating the ratio of the methylated (M) probe signal intensity to the sum of both methylated (M) and unmethylated (U) probe signal intensities. β-values range from 0 to 1, corresponding to completely unmethylated and fully methylated loci, respectively. Prior to normalization, probes with p-value>0.01 in any sample and probes overlapping with single nucleotide polymorphisms (SNPs) in the probe sequence [3] were removed followed by BMIQ normalization [4, 5] and methylumi.noob background subtraction [6]. Subsequently probes outside of CpG context as well as probes on sex chromosomes were removed. Downstream ‘gastric’ data analysis was restricted to the remaining 424383 probes (GSE97686), whereas ‘oesophageal’ data analysis was restricted to 424355 probes (GSE97687). Differential methylation analysis between gastric and oesophageal CAM vs ATM, gastric CAM vs NTM and gastric ATM vs NTM was conducted using the limma method [7]. CpG sites with |Δβ|>0.2, p-value<0.05 were considered differentially methylated.

**Illumina HT-12 Expression data processing and analysis**

To quantify gene expression profiles in stromal myofibroblasts, Illumina HumanHT-12v4 Expression BeadChip arrays were perform on a collection of primary gastric patient-matched CAM and ATM (n=3), and unrelated NTM samples (n=3). The Bioconductor package lumi (version 2.18.0) [8] was used to import the raw data into R. Background correction, variance stabilization transformation, robust spline normalization and subsequent quality control were then performed using this package. To assess the reproducibility of sample preparation, a biological replicate of one of the samples was included (R2=0.9933573). Probes with a detection p-value < 0.01 across all samples were considered non-detectable thus were removed from subsequent analysis. The un-annotated probes were also removed, restricting the analysis to 18090 probes (corresponding to 13381 genes) (GSE107161). To identify differentially expressed transcripts between relevant sample groups (CAM vs ATM, CAM vs NTM and ATM vs NTM) limma package [7] was used. To control for false discovery rate the Benjamini and Hochberg method [9] was applied. Probes with p-value < 0.05 were considered differentially expressed.

**Gene ontology enrichment analysis**

Gene ontology (GO) enrichment analysis was performed on (i) differentially methylated CpG loci identified in gastric and oesophageal CAMs and ATMs, and (ii) differentially expressed genes identified in gastric CAM vs ATM. The GO enrichment analysis on methylation data was conducted using gometh() function from the Bioconductor package missMethyl [10, 11], as this function was designed explicitly for Illumina450k data, taking into account the number of probes per gene on the array. The background was set to 424383 and 424355 probes, respectively. The GO enrichment analysis on gene expression data was performed using GOrilla (Gene Ontology enRIchment anaLysis and visuaLizAtion tool) [12]. Target genes were compared to background gene set of 13381 genes expressed in myofibroblast cells.

**Ingenuity Pathway Analysis**

Ingenuity Pathway Analysis (IPA) software was used to assess biological relevance of differentially methylated genes identified in CAMs. The lists of differentially methylated genes identified in CAM vs ATM and CAM vs NTM comparisons were uploaded to IPA and compared against the Ingenuity Knowledge Base (IKB). IPA canonical pathway and downstream effects analyses were performed, p-value was assigned using Fisher’s exact test which indicates the probability of overlap between the pathway/phenotype and input genes.

**Gene Set Enrichment Analysis**

Gene set enrichment analysis (GSEA v5.0) [13, 14] was performed on differentially expressed genes identified in gastric CAM vs ATM and CAM vs NTM comparisons using the hallmark gene set subcollection (MSigDB v5.0) [14]. The identified gene profiles were separated into two phenotypes for GSEA: CAM and ATM or CAM and NTM. For gene list ranking, multiple probes matching the same gene were sorted according to p-value and the probe with the lowest p-value was retained for the analysis. Genes were ranked using the provided signal-to-noise ranking statistic and GSEA was run using a default weighted enrichment statistics and evaluated for statistical significance by comparison to results obtained using 1 000 random permutations of each gene set. Default settings were used for all other GSEA parameters.

**R/Bioconductor**

R statistical software (version 3.1.2) and Bioconductor [15, 16] were used to process and analyse the Illumina Infinium HumanMethylation450 BeadChip and Illumina HumanHT-12v4 Expression BeadChip arrays.

**AVAILABILITY OF DATA AND MATERIALS**

Gastric and oesophageal DNA methylation data and gastric gene expression data have been deposited in Gene Expression Omnibus (GEO) under accession number GSE97686, GSE97687 and GSE107161, respectively.

**REFERENCES**

1. Bibikova, M., et al., *High density DNA methylation array with single CpG site resolution.* Genomics, 2011. **98**(4): p. 288-295.

2. Assenov, Y., et al., *Comprehensive analysis of DNA methylation data with RnBeads.* Nature methods, 2014. **11**(11): p. 1138-40.

3. Chen, Y.-a., et al., *Discovery of cross-reactive probes and polymorphic CpGs in the Illumina Infinium HumanMethylation450 microarray.* Epigenetics, 2013. **8**(2): p. 203-209.

4. Teschendorff, A.E., et al., *A beta-mixture quantile normalization method for correcting probe design bias in Illumina Infinium 450 k DNA methylation data.* Bioinformatics, 2013. **29**(2): p. 189-196.

5. Wu, M.C., et al., *A systematic assessment of normalization approaches for the Infinium 450K methylation platform.* Epigenetics, 2014. **9**(2): p. 318-329.

6. Triche, T.J., Jr., et al., *Low-level processing of Illumina Infinium DNA Methylation BeadArrays.* Nucleic Acids Research, 2013. **41**(7).

7. Smyth, G.K., *Limma: linear models for microarray data* in *Bioinformatics and Computational Biology Solutions Using R and Bioconductor*, C.V. Gentleman R, Huber W, Irizarry R, Dudoit S (eds.), Editor. 2005, Springer: New York. p. pp. 397-420.

8. Du, P., W.A. Kibbe, and S.M. Lin, *lumi: a pipeline for processing Illumina microarray.* Bioinformatics, 2008. **24**(13): p. 1547-1548.

9. Benjamini, Y. and Y. Hochberg, *Controlling the false discovery rate a practical and powerful approach to multiple testing.* Journal of the Royal Statistical Society Series B-Methodological, 1995. **57**(1): p. 289-300.

10. Maksimovic, J., L. Gordon, and A. Oshlack, *SWAN: Subset-quantile within array normalization for illumina infinium HumanMethylation450 BeadChips.* Genome biology, 2012. **13**(6).

11. Phipson, B. and A. Oshlack, *DiffVar: a new method for detecting differential variability with application to methylation in cancer and aging.* Genome biology, 2014. **15**(9): p. 465.

12. Eden, E., et al., *GOrilla: a tool for discovery and visualization of enriched GO terms in ranked gene lists.* Bmc Bioinformatics, 2009. **10**.

13. Mootha, V.K., et al., *PGC-1alpha-responsive genes involved in oxidative phosphorylation are coordinately downregulated in human diabetes.* Nature genetics, 2003. **34**(3): p. 267-273.

14. Subramanian, A., et al., *Gene set enrichment analysis: a knowledge-based approach for interpreting genome-wide expression profiles.* Proceedings of the National Academy of Sciences of the United States of America, 2005. **102**(43): p. 15545-15550.

15. Gentleman, R.C., et al., *Bioconductor: open software development for computational biology and bioinformatics.* Genome biology, 2004. **5**(10).

16. Huber, W., et al., *Orchestrating high-throughput genomic analysis with Bioconductor.* Nature methods, 2015. **12**(2): p. 115-121.
