# Supplementary material for: Integrated omics profiling reveals novel patterns of epigenetic programming in cancer-associated myofibroblasts
Source: Carcinogenesis. 2019 Jan 8;40(4):500–12. doi: 10.1093/carcin/bgz001 (PMC6556705; doi:10.1093/carcin/bgz001)
Supplement: bgz001_suppl_Supplementary_File_S5 [file bgz001_suppl_supplementary_file_s5.docx]

File S5 GSEA results showing a list of significantly enriched hallmark gene sets in CAM and ATM phenotypes. The right-hand column represents a CAM vs NTM projection of gene sets identified in CAM vs ATM (left-hand column) comparison; *NES* – normalized enrichment score; *NA* – gene set not identified as significantly enriched in CAM vs NTM comparison.

|  |  | **CAM vs ATM** | | | | **CAM vs NTM** | | | |
| --- | --- | --- | --- | --- | --- | --- | --- | --- | --- |
| **Phenotype** | **HALLMARK GENE SET NAME** | **SIZE** | **NES** | ***p-value*** | **FDR** | **SIZE** | **NES** | ***p-value*** | **FDR** |
| CAM | HALLMARK_UNFOLDED_PROTEIN_RESPONSE | 26 | 2.98 | 0 | 0 | *NA* | *NA* | *NA* | *NA* |
| CAM | HALLMARK_MTORC1_SIGNALING | 38 | 2.60 | 0 | 0 | *NA* | *NA* | *NA* | *NA* |
| ATM/NTM | HALLMARK_TNFA_SIGNALING_VIA_NFKB | 28 | -2.38 | 0 | 0 | 26 | -2.21 | 0 | 7.48E-04 |
| ATM/NTM | HALLMARK_KRAS_SIGNALING_UP | 20 | -2.33 | 0 | 6.36E-04 | 19 | -1.61 | 3.30E-02 | 7.22E-02 |
| CAM | HALLMARK_GLYCOLYSIS | 27 | 2.14 | 0 | 1.56E-03 | 16 | 1.02 | 4.40E-01 | 4.27E-01 |
| CAM | HALLMARK_EPITHELIAL_MESENCHYMAL_TRANSITION | 28 | 1.85 | 4.99E-03 | 1.25E-02 | 29 | 1.99 | 0 | 1.03E-02 |
| ATM/NTM | HALLMARK_ADIPOGENESIS | 25 | -1.88 | 1.64E-03 | 1.47E-02 | 21 | -1.18 | 2.56E-01 | 3.12E-01 |
| ATM/NTM | HALLMARK_INFLAMMATORY_RESPONSE | 21 | -1.88 | 3.26E-03 | 1.91E-02 | 18 | -2.01 | 0 | 4.33E-03 |
| ATM/NTM | HALLMARK_COMPLEMENT | 17 | -1.81 | 3.38E-03 | 2.06E-02 | 17 | -1.82 | 6.79E-03 | 2.15E-02 |
| ATM/NTM | HALLMARK_APOPTOSIS | 20 | -1.76 | 1.82E-02 | 2.29E-02 | 18 | -1.10 | 3.40E-01 | 3.72E-01 |
| ATM | HALLMARK_INTERFERON_GAMMA_RESPONSE | 18 | -1.76 | 2.02E-02 | 2.62E-02 | *NA* | *NA* | *NA* | *NA* |
| ATM | HALLMARK_XENOBIOTIC_METABOLISM | 23 | -1.71 | 1.46E-02 | 2.79E-02 | *NA* | *NA* | *NA* | *NA* |
| CAM | HALLMARK_HYPOXIA | 30 | 1.60 | 3.67E-02 | 5.08E-02 | 18 | 1.19 | 2.44E-01 | 4.05E-01 |
| CAM | HALLMARK_UV_RESPONSE_DN | 24 | 1.63 | 2.93E-02 | 5.15E-02 | *NA* | *NA* | *NA* | *NA* |
