# Supplementary material for: Integrated omics profiling reveals novel patterns of epigenetic programming in cancer-associated myofibroblasts
Source: Carcinogenesis. 2019 Jan 8;40(4):500–12. doi: 10.1093/carcin/bgz001 (PMC6556705; doi:10.1093/carcin/bgz001)
Supplement: bgz001_suppl_Supplementary_File_S6 [file bgz001_suppl_supplementary_file_s6.docx]

File S6 KEGG and Reactome pathways affected by DNA methylation changes in gastric and oesophageal CAMs.

| Source | Pathway | *p-value* | FDR |
| --- | --- | --- | --- |
| KEGG | Hippo signalling pathway | 1.28E-06 | 3.24E-04 |
|  | Type I diabetes mellitus | 8.69E-05 | 9.13E-03 |
|  | PI3K-Akt signalling pathway | 1.09E-04 | 9.13E-03 |
|  | Glycosaminoglycan biosynthesis - heparan sulfate / heparin | 2.50E-04 | 1.14E-02 |
|  | Glycosaminoglycan biosynthesis - chondroitin sulfate / dermatan sulfate | 2.55E-04 | 1.14E-02 |
|  | Signalling pathways regulating pluripotency of stem cells | 2.70E-04 | 1.14E-02 |
|  | Inflammatory bowel disease (IBD) | 3.26E-04 | 1.17E-02 |
|  | Cell adhesion molecules (CAMs) | 4.05E-04 | 1.27E-02 |
|  | Pathways in cancer | 8.66E-04 | 2.42E-02 |
|  | Rap1 signalling pathway | 1.21E-03 | 3.05E-02 |
|  | Circadian entrainment | 2.33E-03 | 5.33E-02 |
|  | Allograft rejection | 3.28E-03 | 6.41E-02 |
|  | MAPK signalling pathway | 3.40E-03 | 6.41E-02 |
|  | Long-term depression | 3.56E-03 | 6.41E-02 |
|  | Proteoglycans in cancer | 4.26E-03 | 7.15E-02 |
|  | Hedgehog signalling pathway | 6.30E-03 | 9.13E-02 |
|  | Tight junction | 6.56E-03 | 9.13E-02 |
|  | Intestinal immune network for IgA production | 7.00E-03 | 9.13E-02 |
|  | Phagosome | 7.21E-03 | 9.13E-02 |
|  | Wnt signalling pathway | 7.25E-03 | 9.13E-02 |
|  | Inflammatory mediator regulation of TRP channels | 9.73E-03 | 1.17E-01 |
| Reactome | Developmental Biology | 1.39E-06 | 7.14E-04 |
|  | Transcriptional regulation of pluripotent stem cells | 1.04E-05 | 7.14E-04 |
|  | Glycosaminoglycan metabolism | 2.12E-05 | 7.14E-04 |
|  | POU5F1 (OCT4), SOX2, NANOG repress genes related to differentiation | 7.78E-05 | 2.53E-03 |
|  | Axon guidance | 1.22E-04 | 3.82E-03 |
|  | HS-GAG biosynthesis | 6.43E-04 | 1.95E-02 |
|  | Diseases of glycosylation | 1.51E-03 | 4.36E-02 |
|  | Signalling by FGFR mutants | 1.53E-03 | 4.36E-02 |
|  | Regulation of Rheb GTPase activity by AMPK | 2.10E-03 | 5.81E-02 |
|  | IRS-related events triggered by IGF1R | 3.06E-03 | 8.21E-02 |
|  | Fatty acid, triacylglycerol, and ketone body metabolism | 3.52E-03 | 9.17E-02 |
|  | IGF1R signalling cascade | 4.63E-03 | 1.14E-01 |
|  | Signalling by Type 1 Insulin-like Growth Factor 1 Receptor (IGF1R) | 4.63E-03 | 1.14E-01 |
|  | Chondroitin sulfate/dermatan sulfate metabolism | 5.26E-03 | 1.26E-01 |
|  | Translocation of GLUT4 to the plasma membrane | 5.42E-03 | 1.27E-01 |
|  | Signalling by FGFR1 mutants | 5.56E-03 | 1.27E-01 |
|  | Regulation of lipid metabolism by Peroxisome proliferator-activated receptor alpha (PPARalpha) | 6.84E-03 | 1.48E-01 |
|  | Chondroitin sulfate biosynthesis | 6.84E-03 | 1.48E-01 |
|  | Signalling by NOTCH | 7.24E-03 | 1.53E-01 |
|  | Energy dependent regulation of mTOR by LKB1-AMPK | 8.36E-03 | 1.65E-01 |
|  | EPH-Ephrin signalling | 9.22E-03 | 1.65E-01 |
|  | Hedgehog ,off, state | 9.34E-03 | 1.65E-01 |
|  | Hemostasis | 9.64E-03 | 1.65E-01 |
