# Supplementary material for: Integrated omics profiling reveals novel patterns of epigenetic programming in cancer-associated myofibroblasts
Source: Carcinogenesis. 2019 Jan 8;40(4):500–12. doi: 10.1093/carcin/bgz001 (PMC6556705; doi:10.1093/carcin/bgz001)
Supplement: bgz001_suppl_Supplementary_Figures [file bgz001_suppl_supplementary_figures.docx]

Supplementary Information

**Integrated Omics Profiling Reveals Novel Patterns of Epigenetic Programming in Cancer-Associated Myofibroblasts**

Hanna Najgebauer^1^, Triantafillos Liloglou^2^, Puthen V. Jithesh^2^, Olivier T. Giger^1^, Andrea Varro^1,3^, Christopher M. Sanderson^1^

^1^ Department of Cellular and Molecular Physiology, University of Liverpool, Crown Street, Liverpool L69 3BX, UK

^2^ Department of Molecular and Clinical Cancer Medicine, University of Liverpool, Crown Street, Liverpool L69 3BX, UK

^3^ Department of Medicine, University of Szeged, Hungary

SUPPLEMENTARY FIGURES AND TABLES

Figure S1 Increased migration of gastric cancer cells in response to CAM conditioned media (CM) compared to ATM-CM and NTM-CM. **A.** Representative Boyden chamber migration assays of AGS cells treated with CAM, ATM or NTM conditioned media**. B.** Individual patient-paired Boyden chamber migration assays of AGS cells treated with CAM-CM or ATM-CM. All data points were corrected for basal migration by subtracting respective serum-free media controls. **C.** Group mean data of AGS cell migration in response to CAM-CM (n=4), ATM-CM (n=4) or NTM-CM (n=2). Mean, SEM *** ANOVA p-value < 0.01 (p=0.0093).*

Figure S2 Increased proliferation of gastric cancer cells in response to CAM conditioned media (CM) compared to ATM-CM and NTM-CM. **A.** Individual patient-paired EdU proliferation assays of AGS cells treated with CAM-CM or ATM-CM. All data points were corrected for basal proliferation by subtracting respective serum-free media controls. **B.** Group mean data of AGS cell proliferation in response to CAM-CM (n=3), ATM-CM (n=3) or NTM-CM (n=2). Mean, SEM **paired t-test p-value<0.05 (p=0.0143).*

**Figure S3** DNA methylation in CAMs derived from different tumour types. A, B. Global DNA methylation of gastric and oesophageal CAMs and corresponding patient-matched ATMs. Boxplots represent mean β**-value for CAMs (n=3) and ATMs (n=3)** isolated from: A. gastric cancer (mean β for 424383 CpG sites) and B. oesophageal cancer (mean β for 424355 CpG sites); *Wilcoxon test p-value < 2.2 x10^-16^.* C. Unsupervised clustering of 3611 CpG loci with marked differential methylation in oesophageal CAMs and patient-matched ATMs. |Δβ|>0.2, *p-value <0.05*.

Figure S4 Heatmap representations of CpG loci that may serve as proxies for gastric CAM, ATM and NTM identification. CAM–specific methylation pattern identified in CAM vs ATM and CAM vs NTM |Δβ|>0.2 comparisons.

Figure S5 Correlations between Illumina 450k array data and pyrosequencing analysis in gastric myofibroblasts; *magenta–CAMs, purple–ATMs, navy–NTMs.* Representative data for 12 single CpG site identified as differentially methylated by Ilumina 450k array and validated by pyrosequencing analysis are shown.

Figure S6 Gene ontology (GO) cellular component (CC) enrichment for differentially expressed genes identified in gastric CAM vs ATM comparison. DAG tree represent GO_CC terms relations; colors represent GO term *p-value* as shown in *p-value color scale* legend on top.


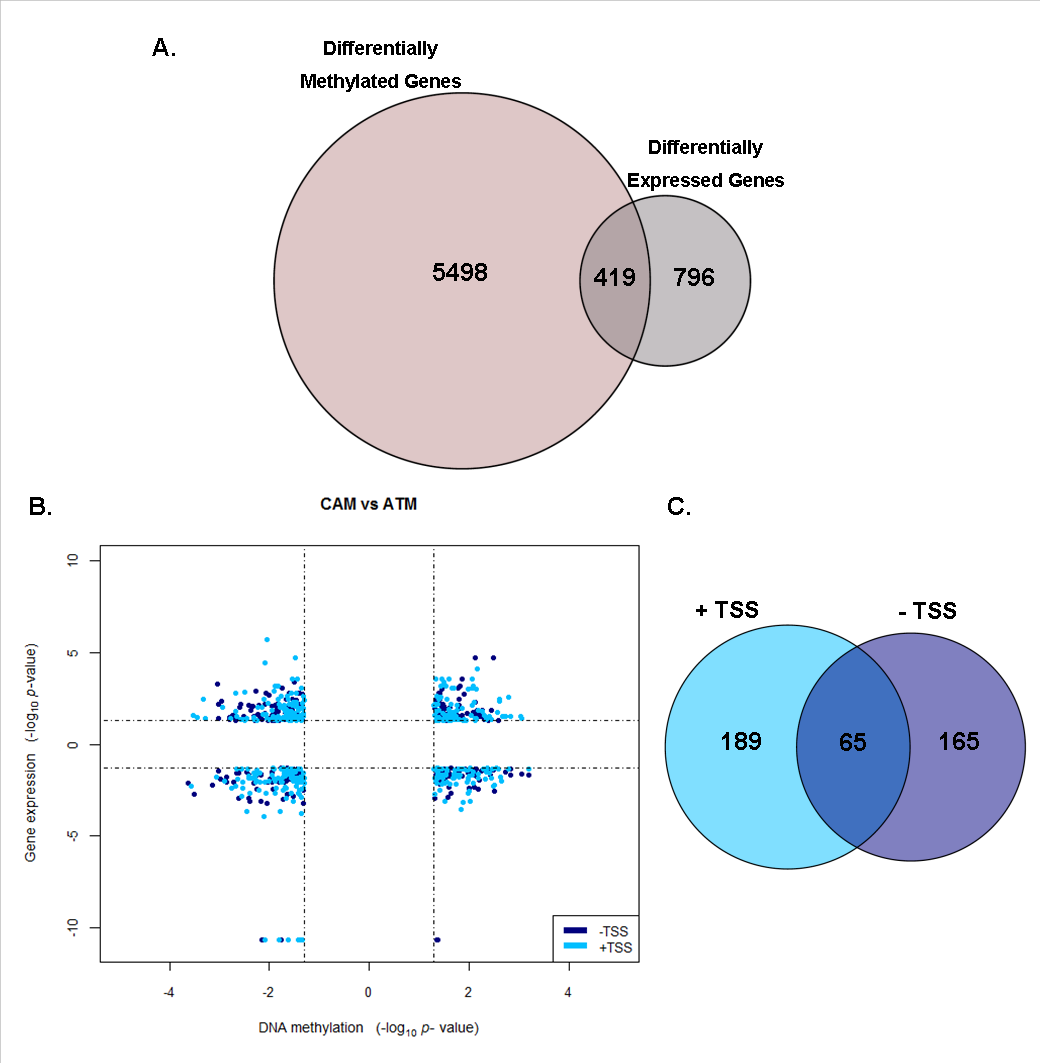


Figure S7 Identification of a subset of genes, which expression may be regulated by DNA methylation changes in gastric CAMs. **A.** Overlap of genes identified as differentially methylated and differentially expressed in gastric CAM vs ATM comparisons. **B.** Quadrant plot for genes showing changes in expression and DNA methylation in CAMs compared to ATMs. Vertical and horizontal dashed lines indicate *p-value < 0.05*. The four quadrants shown are: (i) top left – hypomethylated and upregulated in CAMs, (ii) top right – hypermethylated and upregulated in CAMs, (iii) bottom left – hypomethylated and downregulated in CAMs, (iv) bottom right – hypermethylated and downregulated in CAMs; *navy* - CpG sites downstream of transcription start site (-TSS); *blue* – CpG sites upstream of transcription start site (+TSS). **C.** Venn diagram representing differentially expressed genes that are regulated by changes in DNA methylation upstream (+TSS) and/or downstream (-TSS) of the transcription start site.

Figure S8 Differentially methylated genes in gastric and oesophageal CAMs. **Area-proportional Venn diagram of genes that were associated with differentially methylated CpG loci identified in gastric and oesophageal CAM vs ATM comparisons;** |Δβ|>0.2, *p-value <0.05.*


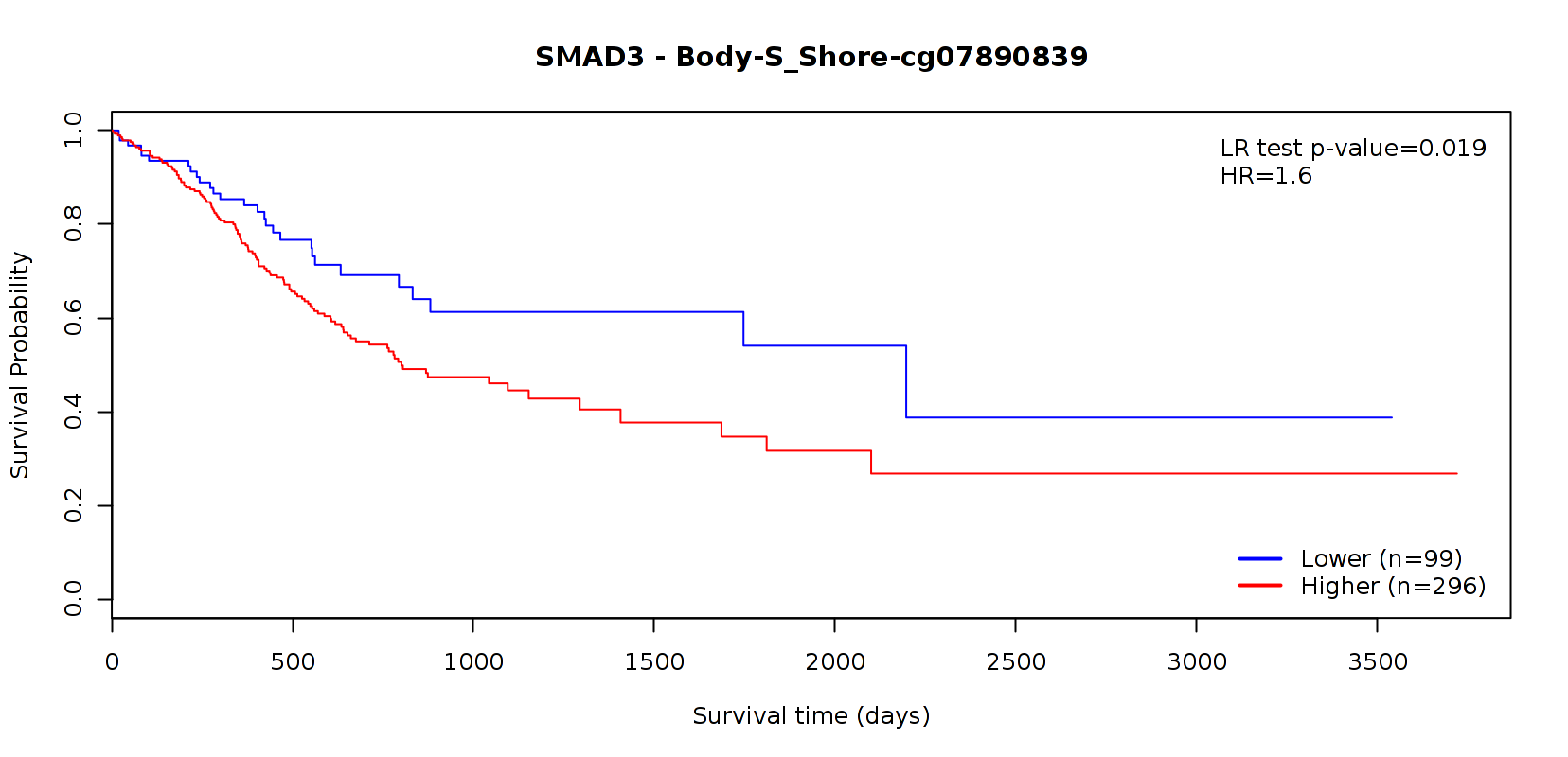


**Figure S9 Kaplan–Meier plot for cg07890839-SMAD3 methylation in TCGA stomach adenocarcinoma (STAD) samples** showing survival in higher β (n=296; shown in red) and lower β (n=99; shown in blue) methylation groups dichotomized by upper quantile (0.25 quantile). The X-axis denotes survival time in days and the Y-axis denotes the probability of patient survival. MethSurv (Modhukur, Iljasenko et al. 2018) was used to obtained TCGA DNA methylation data for STAD samples and to perform the survival analysis.

**Figure S10 SPON2 abundance in gastric CAMs and NTMs based on secretome data.** Comparative CAM (n=3) vs NTM (n=3) secretome profiling showed that SPON2 protein is upregulated 3-fold in the CAM secretome compared to the NTM secretome. Mean, SD.

Figure S11 Comparative DNA methylation profiles within a genomic region on chromosome 16 spanning 9,673bp identified by Illumina 450k array in oesophageal patient-matched CAM and ATM samples. Mean β-values (n=3) for probes identified as differentially methylated in oesophageal CAM vs ATM comparison are plotted. The *X*-axis indicates distance of Illumina 450k probes to the *FOXF1* transcription start site.

Figure S12 Hypermethylation of the FOXF1 promoter region in gastric cancer. A. Pyrosequencing analysis of the FOXF1 promoter region in gastric cancer cell lines. Methylation means for 8 individual CpG sites in interrogated promoter region are plotted for AGS cells (n=3) and MKN45 cells (n=2). The *X*-axis indicates chromosomal position of examined CpG sites. Error bars represent SEM. **B.** Differential DNA methylation data for stomach adenocarcinoma from the Cancer Genome Atlas (TCGA). The presented region corresponds to the differentially methylated region identified in gastric CAMs compared to either ATMs or NTMs as shown in Figure 5A. Mean β values for stomach tumour (n=339; red line) and normal tissue (n=2; blue line) are plotted. The *X-axis* represents the Illumina 450k probes. TCGA data was obtained using Wanderer (Diez-Villanueva, Mallona et al. 2015).

**Table S1** Patient information relating to age, gender, tumour location and clinical assessment of gastric and oesophageal cancer patients used in this study.

| **Patient ID** | **Age** | **Gender** | **Location of Tumour** | **Classification** | **H.pylori status** | **Tumour Staging** | **Survival (months)** | **Adjacent Tissue** |
| --- | --- | --- | --- | --- | --- | --- | --- | --- |
| 1 | 49 | F | antrum corpus border | adenocarcinoma, diffuse | + | pT3N1M0 | 22 | chronic gastritis |
| 2 | 51 | M | antrum corpus border | adenocarcinoma, mixed | + | pT1N3M0 | 9 | chronic gastritis |
| 3 | 59 | F | antrum and corpus | adenocarcinoma, diffuse | + | pT3N2M0 | 17 | chronic gastritis |
| 4 | 72 | M | antrum corpus border | adenocarcinoma, medullar (non-Lauren) | - | pT1N0M0 | 75 | intestinal metaplasia, atrophy |
| 5 | 82 | M | antrum | adenocarcinoma, intestinal | - | pT4N2M0 | 2 | intestinal metaplasia,  chronic gastritis |
| 6 | 72 | M | corpus | adenocarcinoma, mixed | - | pT3N1M0 | 24 | chronic gastritis |
| 7 | 76 | M | antrum | adenocarcinoma, intestinal | + | pT4N2M0 | 15 | intestinal metaplasia, atrophy, chronic gastritis |
| 8 | 72 | M | esophagus | adenocarcinoma | N/A | pT3N1M0 | 18 | Barrett’s eosophagus |
| 9 | 70 | F | esophagus cardia junction | adenocarcinoma | N/A | pT3N1M0 | >25 | Intestinal metaplasia |
| 10 | 64 | M | esophagus cardia junction | adenocarcinoma | N/A | pT2N3M0 | 19 | Intestinal metaplasia |

Table S2 Information relating to origin of tissue, age, and gender of post-mortem organ donors who provided gastric NTMs used in this study.

| **Sample ID** | **Origin** | **Age** | **Gender** |
| --- | --- | --- | --- |
| Donor 1 | gastric corpus | 67 | M |
| Donor 2 | gastric antrum | 60 | M |
| Donor 3 | gastric antrum | 52 | F |
| Donor 4 | gastric antrum | 52 | F |

**Table S3** Nucleotide sequences of PCR primer sets and sequencing primers used for pyrosequencing DNA methylation assays; *NA* – no CpG loci corresponding to Illumina 450k probe is interrogated by the pyrosequencing assay.

| **450k probe ID** | **Sequence 5’ -> 3’** | **Primers ratio** | **Annealing temp** |
| --- | --- | --- | --- |
| cg06472341 | R: 5’-biotin-CAAACCCTAAAACCTAATACTAAC-3’ | 1:2 | 54°C |
|  | F: 5’-TGTTGTTTAGATTTAGGGTTTG-3’ |  |  |
|  | S: 5’-TTAGATTTAGGGTTTGGGA-3’ |  |  |
| cg00386405 | R: 5’-biotin-CAAAACTAACCTCAACAAAATC-3’ | 1:2 | 54°C |
|  | F: 5’-GTTTATAAGTGGTTTGTTGGG-3’ |  |  |
|  | S: 5’-AAGTGGTTTGTTGGGAG-3’ |  |  |
| cg14658493 | R: 5’-GTCAAATTCCATACACTTTTATAACC-3’ | 1:2 | 57°C |
|  | F: 5’-biotin-GATGAAGATTTTTTAGGTTGGATA-3’ |  |  |
|  | S: 5’-ATTCCATACACTTTTATAAC-3’ |  |  |
| cg00871610 | R: 5’-CTCTACAACCTCTTATATCATTTCA-3’ | 1:2 | 54°C |
|  | F: 5’-biotin-GATGAATTTTTGTTATTGATTGTA-3’ |  |  |
|  | S: 5’-AAAAATACCATATACCCATTA-3’ |  |  |
| cg07926952  cg13380112 | R: 5’-biotin-AAAATAATTCTCTCTAACTCCTC-3’ | 1:2 | 52°C |
|  | F: 5’-GTATTTGTAGTTTTGGAGAAAG-3’ |  |  |
|  | S: 5’-TTTGTAGTTTTGGAGAAAGT-3’ |  |  |
| cg23543318 | R: 5’-ACCATCCCCACTCATCT-3’ | 1:1 | 53°C |
|  | F: 5’-biotin-TGTTTGATGTTTTGTTGTGG-3’ |  |  |
|  | S: 5’-CATCCCCACTCATCTCA-3’ |  |  |
| cg11741004 | R: 5’-biotin-TAAACATTATTACCTATAAACACC-3’ | 1:2 | 51°C |
|  | F: 5’-GAATAATTTTGTTGGTGGG-3’ |  |  |
|  | S: 5’-GGTGGGGATGTGTTGGATA-3’ |  |  |
| cg23731272 | R: 5’-biotin-ATTTTCAAAAACTACTCCAAA-3’ | 1:1 | 52°C |
|  | F: 5’-GGGAGATTTTTGTTGTTAAA-3’ |  |  |
|  | S: 5’-ATAAAGGGTTTAGATAT-3’ |  |  |
| cg02486855 | R: 5’-CAACAACAAACCAATTAACA-3’ | 1:2 | 53°C |
|  | F: 5’-biotin-GAGGGGTTTGGAGTAGTT-3’ |  |  |
|  | S: 5’-AACAACAAACCAATTAACAC-3’ |  |  |
| cg02930996  cg07027513 | R: 5’-biotin-ACCTAAATTAATAAATCCCC-3’ | 1:2 | 52°C |
|  | F: 5’-TGAAGGTAAGTTTTGGTATAAG-3’ |  |  |
|  | S: 5’-TAAGTTTTGGTATAAGG-3’ |  |  |
| *NA* | R: 5’-biotin-CCCAAACCTATAACCTCCAC-3’ | 1:2 | 54°C |
|  | F: 5’-GGGAAAAATTTGAGAATAGATAG-3’ |  |  |
|  | S: 5’-TTTGAGAATAGATAGGGG-3’ |  |  |

**Table S4** List of TaqMan assays and nucleotide sequences used in this study.

| **Gene** | **TaqMan Assay ID** | **Amplicon Length (bp)** | **Forward Primer *5’ -> 3’*** | **Reverse Primer *5’ -> 3’*** | **Probe *5’-> 3’*** |
| --- | --- | --- | --- | --- | --- |
| FOXF1 | Hs00230962_m1 | 69 | Proprietary sequence Life Technologies | Proprietary sequence Life Technologies | (FAM) |
| SMAD3 | Hs00969210_m1 | 87 | Proprietary sequence Life Technologies | Proprietary sequence Life Technologies | (FAM) |
| SPON2 | Hs00202813_m1 | 104 | Proprietary sequence Life Technologies | Proprietary sequence Life Technologies | (FAM) |
| FENDRR v1 | custom | 92 | AAGTGAAATACATGTAGATGGGAT | TGTGCCAAACTGAGTAAACC | CACCCTCTCTGGTCTTCAGTTTCTCA (FAM) |
| FENDRR v2 | custom | 111 | GCTTCTGTCCAAGGCACT | CAAGCTTGCTAACTTCTTTGC | AGCCTACTCGTCAAAAGCCCGA (TAMRA) |
| ACTB (TAMRA) | custom | 128 | GGCACCCAGCACAATGAAG | CATACTCCTGCTTGCTGATCCA | CTCCTCCTGAGCGCAAGTACTCCGTG (TAMRA) |
| ACTB (VIC) | 4326315E | 171 | Proprietary sequence Life Technologies | Proprietary sequence Life Technologies | (VIC) |

Table S5 Genes with coordinated changes in promoter methylation and gene expression identified in gastric CAM vs ATM comparisons.

|  |  | |  | | Expression | | | | | Methylation | | |  |
| --- | --- | --- | --- | --- | --- | --- | --- | --- | --- | --- | --- | --- | --- |
| Gene Symbol | **HT-12 probe** | | **450k probe** | | **Log_2_ FC** | **FC** | | ***p-value*** | | **CAM β** | **ATM β** | ***p - value*** | ***Spearman correlation*** |
| STOM | ILMN_1696419 | | cg14215970 | | -0.943 | 0.520 | | 4.72E-03 | | 0.791 | 0.590 | 2.51E-02 | -0.257 |
| SEPP1 | ILMN_1785071 | | cg12887985 | | -0.830 | 0.563 | | 4.60E-02 | | 0.870 | 0.620 | 3.25E-03 | -0.657 |
| MGAT3 | ILMN_1853824 | | cg03318904 | | -0.806 | 0.572 | | 1.18E-02 | | 0.446 | 0.181 | 6.23E-03 | -0.029 |
| TBX2 | ILMN_1792256 | | cg19457909 | | -0.726 | 0.605 | | 2.75E-02 | | 0.866 | 0.235 | 2.85E-02 | -0.943 |
| WNT2B | ILMN_1740269 | | cg04571584 | | -0.710 | 0.611 | | 4.87E-02 | | 0.912 | 0.577 | 1.49E-03 | -0.429 |
| ZNF536 | ILMN_2150586 | | cg00386405 | | -0.697 | 0.617 | | 2.32E-11 | | 0.626 | 0.358 | 4.25E-02 | -0.886 |
|  | ILMN_2150586 | | cg05804948 | | -0.697 | 0.617 | | 2.32E-11 | | 0.322 | 0.014 | 4.42E-02 | -0.886 |
| SPRY1 | ILMN_2329914 | | cg00137840 | | -0.590 | 0.664 | | 2.73E-02 | | 0.910 | 0.691 | 7.61E-03 | -0.257 |
| TGFBR2 | ILMN_2384241 | | cg00807684 | | -0.587 | 0.666 | | 2.38E-02 | | 0.809 | 0.292 | 8.59E-04 | -0.257 |
|  | ILMN_2384241 | | cg19142043 | | -0.587 | 0.666 | | 2.38E-02 | | 0.962 | 0.700 | 2.02E-02 | -0.600 |
| FAT1 | ILMN_3247578 | | cg03199366 | | -0.516 | 0.699 | | 2.79E-02 | | 0.717 | 0.456 | 1.05E-02 | -0.314 |
| ABR | ILMN_1672878 | | cg19807420 | | -0.452 | 0.731 | | 4.74E-02 | | 0.879 | 0.531 | 1.73E-02 | -0.429 |
|  | ILMN_1672878 | | cg05856321 | | -0.452 | 0.731 | | 4.74E-02 | | 0.843 | 0.356 | 2.97E-02 | -0.429 |
| SULF1 | | ILMN_1702363 | | cg10791884 | 1.051 | | 2.072 | | 6.87E-03 | 0.050 | 0.258 | 9.70E-04 | -0.600 |
|  |  | ILMN_1702363 | | cg18545695 | 1.051 | | 2.072 | | 6.87E-03 | 0.055 | 0.394 | 1.42E-02 | -0.600 |
| SLC6A9 | | ILMN_1714445 | | cg25387812 | 0.847 | | 1.799 | | 5.05E-04 | 0.597 | 0.891 | 8.99E-04 | -0.829 |
| SGCG | | ILMN_1659649 | | cg00748494 | 0.815 | | 1.759 | | 3.23E-02 | 0.060 | 0.271 | 8.89E-03 | -0.886 |
|  |  | ILMN_1659649 | | cg11067829 | 0.815 | | 1.759 | | 3.23E-02 | 0.331 | 0.603 | 1.40E-02 | -0.943 |
|  |  | ILMN_1659649 | | cg07126559 | 0.815 | | 1.759 | | 3.23E-02 | 0.279 | 0.557 | 2.27E-02 | -0.543 |
| SPARC | | ILMN_1796734 | | cg19939793 | 0.669 | | 1.590 | | 7.83E-03 | 0.055 | 0.322 | 6.04E-03 | -0.543 |
| SPON2 | | ILMN_1676099 | | cg13558774 | 0.662 | | 1.582 | | 6.27E-03 | 0.107 | 0.653 | 2.69E-03 | -0.886 |
|  |  | ILMN_1676099 | | cg23543318 | 0.662 | | 1.582 | | 6.27E-03 | 0.032 | 0.288 | 8.08E-03 | -0.600 |
|  |  | ILMN_1676099 | | cg13653809 | 0.662 | | 1.582 | | 6.27E-03 | 0.431 | 0.788 | 3.64E-02 | -0.714 |
|  |  | ILMN_1676099 | | cg23127323 | 0.662 | | 1.582 | | 6.27E-03 | 0.174 | 0.622 | 3.73E-02 | -0.600 |
| COL5A1 | | ILMN_1706505 | | cg13478045 | 0.622 | | 1.539 | | 1.60E-03 | 0.358 | 0.777 | 2.53E-02 | -0.829 |
|  |  | ILMN_1706505 | | cg00753924 | 0.622 | | 1.539 | | 1.60E-03 | 0.084 | 0.292 | 3.93E-02 | -0.257 |
| SEL1L3 | | ILMN_1797822 | | cg25546651 | 0.614 | | 1.530 | | 1.02E-02 | 0.039 | 0.356 | 2.21E-02 | -0.543 |
| SYNM | | ILMN_1712075 | | cg26942432 | 0.515 | | 1.429 | | 2.36E-02 | 0.625 | 0.953 | 1.33E-02 | -0.600 |
| CHAC1 | | ILMN_1739241 | | cg07891971 | 0.477 | | 1.392 | | 3.87E-04 | 0.116 | 0.317 | 3.16E-02 | -0.543 |
| CERCAM | | ILMN_1750563 | | cg21205865 | 0.458 | | 1.374 | | 1.90E-02 | 0.460 | 0.743 | 2.08E-02 | -0.886 |

Table S6 Genes with coordinated changes in gene-body methylation and gene expression identified in gastric CAM vs ATM comparisons.

|  |  |  | Expression | | | Methylation | | |  |
| --- | --- | --- | --- | --- | --- | --- | --- | --- | --- |
| Gene Symbol | **HT-12 probe** | **450k probe** | **Log_2_ FC** | **FC** | ***p - value*** | **CAM β** | **ATM β** | ***p - value*** | ***Spearman correlation*** |
| AKR1B1 | ILMN_1701731 | cg26925463 | -1.145 | 0.452 | 3.96E-03 | 0.276 | 0.760 | 1.76E-03 | 0.771 |
| LIMCH1 | ILMN_2139761 | cg03742238 | -1.118 | 0.461 | 2.15E-02 | 0.027 | 0.308 | 1.13E-02 | 0.600 |
| ABCC4 | ILMN_2194009 | cg15896117 | -0.978 | 0.508 | 1.50E-03 | 0.667 | 0.891 | 2.81E-03 | 0.657 |
| RSPO3 | ILMN_1681983 | cg10997634 | -0.922 | 0.528 | 9.93E-03 | 0.542 | 0.858 | 1.00E-02 | 0.657 |
| CD248 | ILMN_1726589 | cg08924469 | -0.884 | 0.542 | 1.77E-02 | 0.624 | 0.840 | 3.01E-02 | 0.829 |
| LAMA5 | ILMN_1773567 | cg04632997 | -0.853 | 0.554 | 8.12E-03 | 0.487 | 0.717 | 4.99E-02 | 0.886 |
| RASIP1 | ILMN_1755657 | cg02804819 | -0.852 | 0.554 | 8.39E-03 | 0.107 | 0.478 | 6.39E-03 | 0.486 |
|  | ILMN_1755657 | cg19420129 | -0.852 | 0.554 | 8.39E-03 | 0.240 | 0.522 | 1.71E-02 | 0.943 |
|  | ILMN_1755657 | cg07611790 | -0.852 | 0.554 | 8.39E-03 | 0.087 | 0.531 | 1.84E-02 | 0.600 |
| RFTN1 | ILMN_1800787 | cg01336231 | -0.847 | 0.556 | 1.22E-02 | 0.143 | 0.624 | 5.15E-03 | 0.543 |
| PPARG | ILMN_1800225 | cg07676920 | -0.733 | 0.602 | 1.23E-03 | 0.675 | 0.901 | 2.22E-02 | 0.943 |
| DENND2A | ILMN_1666503 | cg11176169 | -0.732 | 0.602 | 3.29E-02 | 0.342 | 0.554 | 2.14E-02 | 0.257 |
| PSAT1 | ILMN_1692938 | cg13612583 | 2.029 | 4.080 | 7.99E-05 | 0.846 | 0.592 | 6.85E-03 | 0.886 |
| SLC7A5 | ILMN_1720373 | cg26569315 | 1.230 | 2.346 | 2.55E-04 | 0.746 | 0.422 | 3.46E-02 | 0.657 |
| KLF2 | ILMN_1735930 | cg19280540 | 0.902 | 1.869 | 7.14E-03 | 0.819 | 0.613 | 1.73E-02 | 0.257 |
|  | ILMN_1735930 | cg02668248 | 0.902 | 1.869 | 7.14E-03 | 0.860 | 0.657 | 4.65E-02 | 0.600 |
| MTHFD1L | ILMN_1772521 | cg00945443 | 0.897 | 1.862 | 8.83E-04 | 0.683 | 0.469 | 8.79E-03 | 0.886 |
| SULF2 | ILMN_1667460 | cg14286048 | 0.872 | 1.831 | 7.18E-03 | 0.530 | 0.226 | 4.59E-02 | 0.429 |
| SLC7A1 | ILMN_1683859 | cg15955521 | 0.828 | 1.775 | 2.89E-04 | 0.811 | 0.608 | 8.49E-03 | 0.943 |
|  | ILMN_1683859 | cg18091046 | 0.828 | 1.775 | 2.89E-04 | 0.897 | 0.314 | 4.62E-02 | 0.943 |
| SHMT2 | ILMN_1661264 | cg13433012 | 0.759 | 1.693 | 8.02E-04 | 0.457 | 0.106 | 2.15E-02 | 0.714 |
| TARS | ILMN_1685480 | cg15902864 | 0.675 | 1.596 | 2.75E-04 | 0.542 | 0.226 | 2.48E-02 | 0.886 |
| SPON2 | ILMN_1676099 | cg08462122 | 0.662 | 1.582 | 6.27E-03 | 0.772 | 0.428 | 4.95E-02 | 0.771 |
| FGFRL1 | ILMN_1795865 | cg20518497 | 0.633 | 1.551 | 6.58E-04 | 0.833 | 0.625 | 8.26E-03 | 0.886 |
|  | ILMN_1795865 | cg10825234 | 0.633 | 1.551 | 6.58E-04 | 0.862 | 0.529 | 2.92E-02 | 0.829 |
|  | ILMN_1795865 | cg01321174 | 0.633 | 1.551 | 6.58E-04 | 0.778 | 0.571 | 3.66E-02 | 0.086 |
